# Possible Effects of Beetroot Supplementation on Physical Performance Through Metabolic, Neuroendocrine, and Antioxidant Mechanisms: A Narrative Review of the Literature

**DOI:** 10.3389/fnut.2021.660150

**Published:** 2021-05-13

**Authors:** Hamid Arazi, Ehsan Eghbali

**Affiliations:** Department of Exercise Physiology, Faculty of Sport Sciences, University of Guilan, Rasht, Iran

**Keywords:** beetroot supplement, resistance exercise, skeletal muscle, nitrate, O_2_ cost, dietary supplements, endurance exercise

## Abstract

Athletes often seek to use dietary supplements to increase performance during exercise. Among various supplements, much attention has been paid to beetroot in recent years. Beetroot is a source of carbohydrates, fiber, protein, minerals, and vitamins; also, it is a natural source of nitrate and associated with improved sports performance. Nitrates can the modification of skeletal muscle contractile proteins or calcium handling after translation. The time to reach the peak plasma nitrate is between 1 and 3 h after consumption of a single dose of nitrate. Nitrate is metabolized by conversion to nitrite and subsequently nitric oxide. Beetroot can have various effects on athletic performance through nitric oxide. Nitric oxide is an intracellular and extracellular messenger for regulating certain cellular functions and causes vasodilation of blood vessels and increases blood flow. Nitric oxide seems to be effective in improving athletic performance by increasing oxygen, glucose, and other nutrients for better muscle fueling. Nitric oxide plays the main role in anabolic hormones, modulates the release of several neurotransmitters and the major mediators of stress involved in the acute hypothalamic-pituitary-adrenal response to exercise. Beetroot is an important source of compounds such as ascorbic acid, carotenoids, phenolic acids, flavonoids, betaline, and highly active phenolics and has high antioxidant properties. Beetroot supplement provides an important source of dietary polyphenols and due to the many health benefits. Phytochemicals of Beetroot through signaling pathways inhibit inflammatory diseases. In this study, the mechanisms responsible for these effects were examined and the research in this regard was reviewed.

## Introduction

In sports competitions, the winning margin is decreasing and, in some cases the results of competitions may change by a fraction of a second due to the performance of athletes. Therefore, athletes are constantly looking for every benefit to improve sport performance. Some athletes maybe resort to dietary supplements (natural and organic resources) to provide these benefits (1). Dietary supplements are a significant option for the elite and recreational athletes to enhance their performance (2). Athletes are exposed to a variety of food products marketed with the claim of improving health, efficiency, and performance. However, few studies confirm these claims, and the affection and safety of these products are questioned (3).

**Figure 1:**
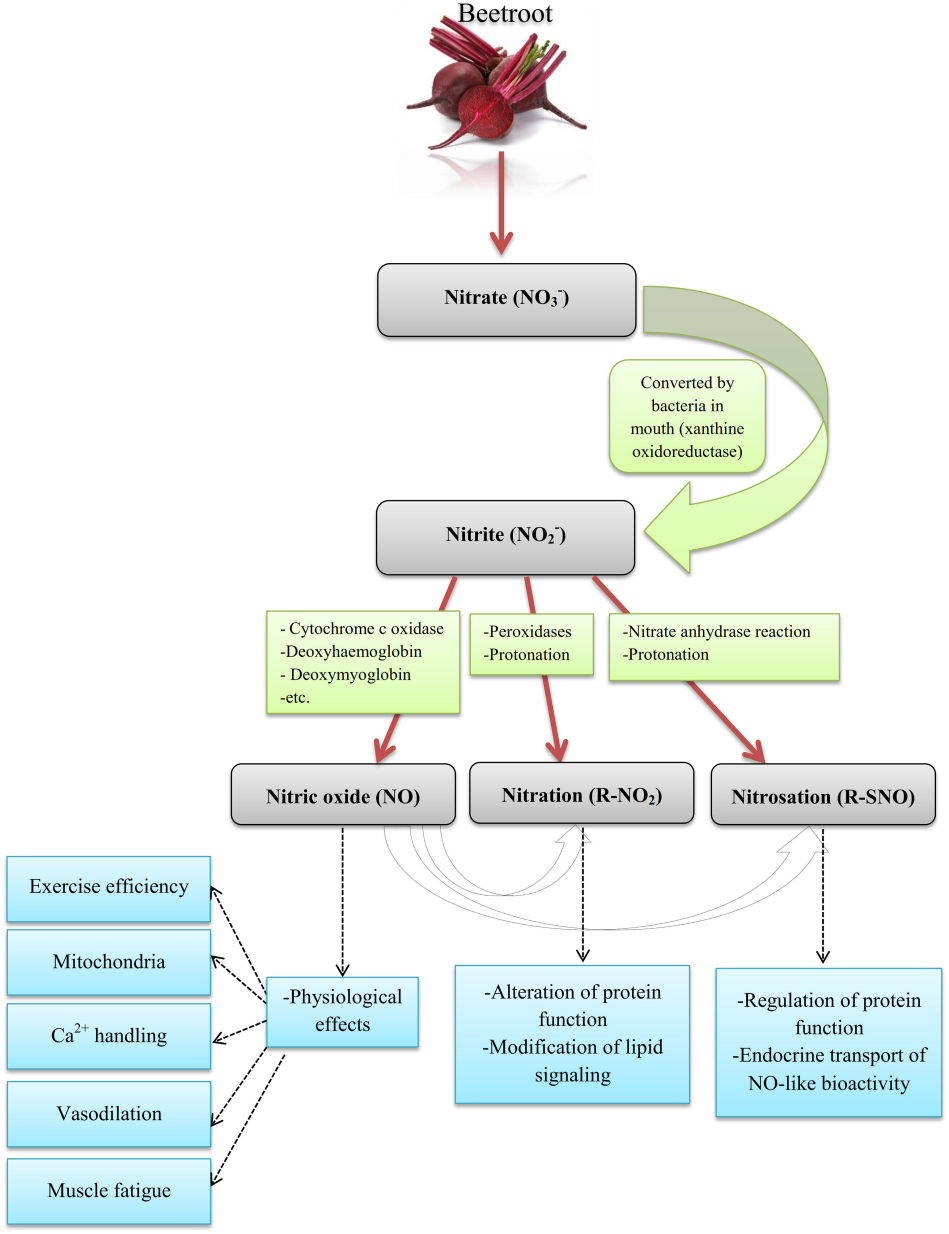
The pathways of NO production from Beetroot in humans. Source: Ormsbee et al. (1); Lundberg et al. (10); Weitzberg and Lundberg (11).

There are many dietary supplements, however in recent years; special attention has been paid to beetroot (BR) supplements. BR is a source of carbohydrates, fiber, protein, vitamins, and minerals (sodium, potassium, calcium, and iron) (as shown in Table 1) (4–8) and among other foods rich in nitrate (${\text{NO}}_{3}^{-}$) including spinach, celery, lettuce, and carrot juice has high ${\text{NO}}_{3}^{-}$ (<250 mg per 100 g of fresh BR) (9). ${\text{NO}}_{3}^{-}$ can be converted to nitrite (${\text{NO}}_{2}^{-}$) by bacteria in the oral cavity and by certain enzymes (e.g., xanthine oxidase) in the tissue (1) and, then the ${\text{NO}}_{2}^{-}$ is swallowed, absorbed in the intestine and nitric oxide (NO) and other active nitrogen oxides are metabolized in the blood and tissues (10, 11) (as shown in Figure 1). ${\text{NO}}_{3}^{-}$ is absorbed through plasma after consumption and its average half-life is 5 h. After absorption into the bloodstream, about 25% return to the salivary glands via active transfer and are concentrated in the saliva, with the remainder exorcized by the kidneys (12–15). Daily doses of 4.1–16.8 mmol (~150 mg to 1 g) of ${\text{NO}}_{3}^{-}$ consumed in 2–15 days enhance the level of ${\text{NO}}_{2}^{-}$ in the blood (12–15). The Jones study showed that the typical mean used in the studies was 5–9 mmol (300–550 mg) (12); ${\text{NO}}_{3}^{-}$ is usually taken between 1.5 and 3 h prior to training in a single dose up to 5 times a day (16–20). The time to reach the peak plasma ${\text{NO}}_{3}^{-}$ is between 1 and 3 h after consumption of a single dose of ${\text{NO}}_{3}^{-}$ (21).

**Table 1:** Nutrient composition of raw Beetroot (per 100 g).

**Nutrients**	**Beetroot (per 100 g)**
Water, g	87.58
Energy, kcal	43
Protein, g	1.61
Total fats, g	0.17
Carbohydrate, g	9.56
Fiber, g	2.8
Sugars, g	6.76
Flavonoid, mg/g	0.41–1.16
Riboflavin, mg	0.04
Betalain, mg	14.20
Carotenoids, mg	1.9
**Minerals**	
Calcium, mg	16
Iron, mg	0.8
Magnesium, mg	23
Phosphorus, mg	40
Potassium, mg	325
Sodium, mg	78
Zinc, mg	0.35
**Vitamins**	
C, mg	4.9
B_1_, mg	0.031
B_2_, mg	0.057
B_3_, mg	0.334
E, mg	0.04
K, mg	0.2
**Essential and non-essential amino acid**	
Tryptophan, g	0.019
Isoleucine, g	0.048
Leucine, g	0.068
Tyrosine, g	0.038
Arginine, g	0.042
Glycine, g	0.031
Alanine, g	0.060
Glutamic acid, g	0.428

*Baiao et al. (4); Chawla et al. (5); Chhikara et al. (6); Kale et al. (7); United States Department of Agriculture Agricultural Research Service. Food Data Central: Food and Nutrient Database for Dietary Studies with Standard Reference Legacy (2019) www.fdc.nal.usda.gov (8)*.

There are several pathways for the metabolism of ${\text{NO}}_{2}^{-}$ to NO and other nitrogen oxides biologically (22). NO is a signaling molecule formed by the endothelial enzyme NO synthase in the endothelium, which causes vasodilatation and increased blood flow (23, 24). NO increases blood flow at rest (25) and during training (26). NO seems to be effective in improving athletic performance by increasing oxygen (O_2_), glucose and other nutrients for better muscle fuel (1). Based on evidence and research on the effects of ${\text{NO}}_{3}^{-}$, the International Olympic Committee (IOC) classified it as a supplement that may improve performance (alongside Creatine and caffeine) (27). Therefore, in this study, we seek to assess the influence of BR supplement consumption on the various dimensions of athletic performance and effective mechanisms.

## Methods

**Figure 2:**
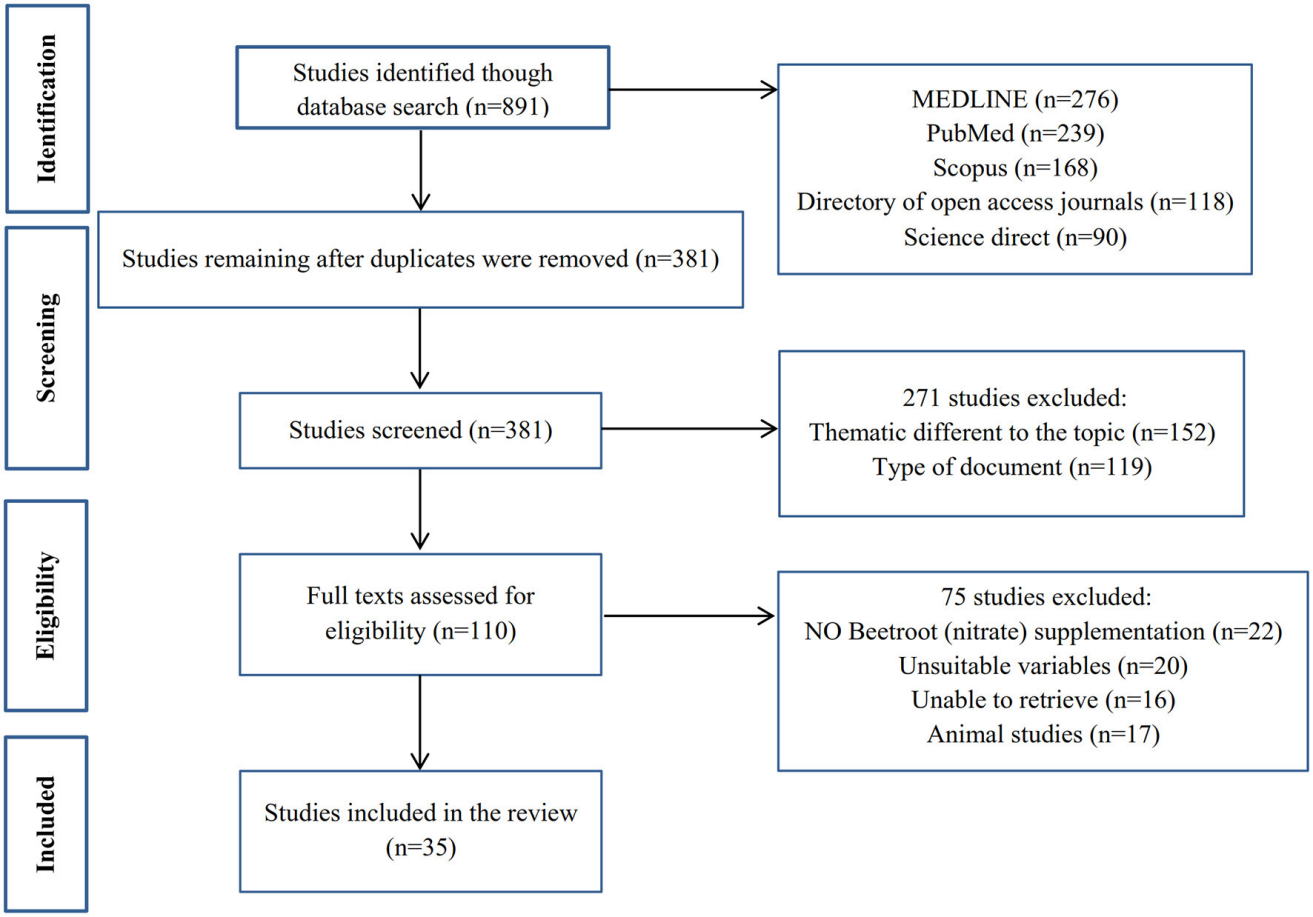
Flow chart of the methodology for the search results.

Based on the purpose of the study, a search was conducted in data bases (MEDLINE, PubMed, Scopus, Directory of open access journals and Science direct databases); the keywords used included Beetroot supplementation, Nitrate supplementation, physical exercise, resistance exercise, aerobic exercise, endurance training, strength training, oxidative stress, O_2_ cost, skeletal muscle, hormonal response, nervous function, and mitochondria. Our focus was on English articles published from 2013 to 2020 (Figure 2).

## The Influence of BR Supplementation on the Skeletal Muscle

BR supplements have received less attention in resistance training (28) (Table 2). In this case, we can refer to the research of Mosher et al. who stated that taking BR supplement for 6 days increases muscle endurance and number of repetitions (32). Moreover, recent studies have shown that BR preferentially enhance blood flow (58) and muscle contraction (59) in the type II muscle fibers, but has no effect on the type 1 fibers. Therefore, BR may be able to increase performance in exercises that use the type 2 muscle fibers (such as resistance training). In addition, a ${\text{NO}}_{3}^{-}$-rich supplement can increase neuromuscular performance during strenuous resistance training (28). Previous evidence suggested that sodium nitrite (NaNO_2_) administration probably enhance cytosolic Ca^2+^ without changing force generation at a supraphysiological partial pressure of oxygen (PO_2_) (60), or decrease cytosolic Ca^2+^ along with less submaximal, but not maximal, force at a physiological PO_2_ (61) (during isometric contractions stimulated in isolated rat muscle fibers). However, during a repetitive and fatigue-stimulating contraction protocol, administration of NaNO_2_ increases t exhaustion time by compensating for decreased Ca^2+^ pumping and Ca^2+^ sensitivity (62).

**Table 2:** Summary of studies investigating the effects of Beetroot supplementation on skeletal muscle, hormonal response, nervous function, O_2_ Cost, mitochondria, and oxidative stress.

**Studies**	**Subject**	**Aim**	**Intervention**	**Main outcome**
**BR supplementation and skeletal muscle**				
Fulford et al. (29)	Healthy, physically active (*N* = 8)	Assess the role of dietary ${\text{NO}}_{3}^{-}$ in regulating force generation under normal physiological conditions	-Received 0.5 l/day of BRJ for 15 days -Exercise protocol: 50 MVCs at 2.5 h, 5, and 15 days after the beginning of the supplement consumption period	↓PCr cost of force production-Improved muscle efficiency
Hoon et al. (30)	Healthy participants (*n =* 18)	Assess the effect of ${\text{NO}}_{3}^{-}$ consumption on muscle contraction	-Days 1–3: 525 mg ${\text{NO}}_{3}^{-}$, day 4: 1,050 mg ${\text{NO}}_{3}^{-}$	=Maximal force, submaximal contractile force-Improved Ca^2+^ handling in the muscle ↓Muscular fatigue
Flanagan et al. (31)	RT men (*n =* 14)	The effects of NR supplement consumption on neuromuscular efficiency	-The NR Bar contained 3 g of concentrated BR extract for 3 days	-Provided neuromuscular advantages during metabolically taxing resistance exercise↑oxygen consumption
Mosher et al. (32)	Recreational active resistance trained males (*n =* 12)	Examine the effects of ${\text{NO}}_{3}^{-}$ consumption on performance of bench press resistance exercise till failure	−6 consecutive days of 70 ml of ${\text{NO}}_{3}^{-}$ shot containing 6.4 mmol/L or 400 mg of ${\text{NO}}_{3}^{-}+$resistance exercise session (60%1RM)	↑Total work and repetitions until failure ↓Energy cost= RPE, blood lactate
Whitfield et al. (33)	Recreationally active males (n = 8)	Investigate the influence of 7 d of BRJ ingestion on skeletal muscle contractile characteristics and function	−7 days of BRJ supplement consumption (280 mL.d^−1^, 26 mmol ${\text{NO}}_{3}^{-}$) - Performed 20 min of cycling (10 min at 50 and 70% VO_2peak_) 48 h before “Pre” and “Post” 5 day of supplement consumption	=Maximal voluntary force production or electrically induced tetanic contractions↑Force production, maximal rates of force development -Change in calcium handling, the content of associated proteins (SERCA1a, SERCA2a, dihydropyradine receptor, ryanodine receptor, and calsequestrin)
de Oliveira et al. (34)	Adult male Brazilian jiu-jitsu trained athletes (*n =* 12)	Investigate the effect of BR-based gel (BG) consumption on MVC, exercise time until fatigue, muscle O_2_ saturation (SmO_2_), blood volume (tHb), and plasma ${\text{NO}}_{3}^{-}$ and lactate in response to handgrip isotonic exercise	−100 g of BR-based nutritional gel containing 12.2 ± 0.2 mmol of ${\text{NO}}_{3}^{-}$, 8 days, 120 min previous exercise	- Prevented force decrease after the handgrip exercise-Improved forearm muscl O_2_ saturation and delayed the accumulation of blood lactate.= Exercise time until fatigue
Papadopoulos et al. (35)	Young males (*n =* 16)	Investigate the effects of BRJ on *in vivo* skeletal muscle VO_2_ and microvascular reactivity at rest and muscle performance, muscle oxygenation during sustained isometric handgrip exercise	-${\text{NO}}_{3}^{-}$ -rich BRJ (500 mg/8.1 mmol ${\text{NO}}_{3}^{-}$, BRJ ${\text{NO}}_{3}^{-}$), After 2.5 h of BRJ consumption participants performed the tests	= Skeletal muscle microvascular reactivity and basal oxidative efficiency↑Muscle oxygenation-Provided ergogenic benefits
Ranchal-Sanchez et al. (28)	Healthy men (*n =* 12)	Examine the acute influence of BRJ on muscular endurance and movement concentric velocity during RT	-Incremental RT test with three sets, at 60, 70, and 80% 1RM. -One of the drinks, 70 mL of BRJ, 120 min before each visit	-Ergogenic effect on the muscular endurance↑Total repetitions performed=RPE
Williams et al. (36)	RT male subjects (*n =* 11)	Assess the effects of acute BRJ ingestion on power, velocity, and repetitions to failure (RTF) during bench press exercise	−70 ml of BRJ, 2 h before exercise	↑Mean velocity and mean power, total RTF.-Positively impacts velocity, power, and total repetitions
Jodra et al. (37)	Resistance trained male (*n =* 15)	Examine the effects of 6 ${\text{NO}}_{3}^{-}$ rich BJ consumption on POMS, RPE, and performance in a 30 s Wingate cycle test	−3 h before initiating Wingate test participant consumed 70 ml of BRJ	↑Peak power output (W_peak_) ↓ Time taken to reach W_peak_↑POMS prior to the Wingate test ↓RPE _muscular_ immediately follows the Wingate test
Jonvik et al. (38)	Recreational active males (*n =* 14)	Examine the effect of BRJ ingestion on maximal isometric strength and isokinetic power, workload achieved during 30 reciprocal voluntary isokinetic contractions and CMJ performance	−140 mL/d ${\text{NO}}_{3}^{-}$ -rich (BR; 985 mg/d), 6-d supplementation periods -Three h following the last supplement, assessed indicators	= Maximal strength, CMJ performance and muscular endurance
Rodríguez-Fernández et al. (39)	Adult males (*n =* 18)	Examine the impact of BRJ consumption on power output during concentric and eccentric muscle contractions during a half-squat	−140 mL dose of 2 × 70 mL concentrated ${\text{NO}}_{3}^{-}$ rich BR shots providing 400 mg ${\text{NO}}_{3}^{-}$/70 mL. Acute ingestion BR 2.5 h before test	↑Mean and peak lower limb power output in the concentric and eccentric movement phases of a half-squat
**BR supplementation and hormonal response**				
Singh et al. (40)	Infantry soldiers (*n =* 30)	Examined the effects of 15 days dietary ${\text{NO}}_{3}^{-}$supplementation on High Density Lipoprotein-Cholesterol and Oxidative Stress in Physically Active Individuals	−400 ml BRJ (consumed twice daily) for 15 days	↓Cortisol levels
Roberts et al. (41)	Healthy non-obese volunteers (*n =* 19)	Assess the impact of inorganic nitrate on markers of the adaptive response to exercise in skeletal muscle	−2 × 70 mL/day BRJ (12 mmol nitrate) for 7 days	↑Circulating growth hormone levels
Garnacho-Castaño et al. (42)	Well-trained CF (*n =* 12)	Asses the causal physiological association between hormonal, metabolic and mechanical responses, and CF workouts performance after acute BJ consumption	-Ingestion 140 mL of BRJ (~12.8 mmol ${\text{NO}}_{3}^{-}$), 3 h before the start of each test (CF workout)	↑Cortisol and testosterone levels
**BR supplementation and nervous function**				
de Vries and DeLorey (43)	Men (*N =* 12)	Investigate the hypothesis that acute dietary ${\text{NO}}_{3}^{-}$ ingestion would attenuate sympathetic vasoconstrictor responsiveness at rest and during exercise	-Consumption of ${\text{NO}}_{3}^{-}$ rich BRJ (~12.9 mmol ${\text{NO}}_{3}^{-}$), Exercise: 2.5 h after consumption	=Plasma catecholamines, and sympathetic vasoconstrictor at rest or during exercise
Kozlowska et al. (44)	Elite fencers (*n =* 20)	Investigate of the long-term metabolic effect of a diet with and without BRJ supplement consumption	−4 weeks with 26 g/d of freeze-dried BRJ consumption	-Significant changes in tyrosine and tryptophan metabolism, mainly associated with such neurotransmitter's metabolism as: serotonin, noradrenaline, and adrenaline
**BR supplementation and O**_**2**_ **Cost and mitochondria**				
Kelly et al. (45)	Healthy subjects (*n =* 12)	Examine the influence of dietary ${\text{NO}}_{3}^{-}$ ingestion on the concentration of plasma ${\text{NO}}_{2}^{-}$, VO_2_ kinetics, and exercise tolerance in normoxia and hypoxia	−140 ml/day of ${\text{NO}}_{3}^{-}$ rich BRJ (8.4 mmol NO3; BR) for 3 days prior to moderate-intensity and severe-intensity exercise tests	↑VO_2_ kinetics- Improving exercise economy and exercise tolerance in hypoxia
Pinna et al. (46)	Trained male master swimmers (*n =* 15)	Investigate whether BRJ supplementation can also improve performance	-Swimming test after 6 days of BRJ (0.5 l/day organic BRJ containing about 5.5 mmol of ${\text{NO}}_{3}^{-}$)	↓Energy cost↑Workload at anaerobic threshold
Muggeridge et al. (18)	Competitive amateur male cyclists (*n =* 9)	Assess the influence of a single dose of BR ingestion on the physiological responses to submaximal exercise and TT performance	-Consumption of either 70 mL of BR, 3 h before exercise	↓VO_2_ during submaximal exercise↑TT performance of trained cyclists in normobaric hypoxia
Arnold et al. (47)	Male runners (*n =* 10)	Investigated the effect of ${\text{NO}}_{3}^{-}$ ingestion upon endurance running performance at altitude in well-trained runners	−7 mmol ${\text{NO}}_{3}^{-}$ at 2.5 h before exercise	= Oxygen cost, arterial oxygen saturation, heart rate, and RPE
MacLeod et al. (48)	Trained male cyclists (*n =* 11)	Assess the influence of BR supplementation on steady-state exercise economy and 10-km TT performance in normoxia and moderate hypoxia (simulated altitude: ~2,500 m)	-Two h before exercise, subjects consumed 70 mL BR (~6 mmol ${\text{NO}}_{3}^{-}$) - VO_2peak_ ≥ 60 ml·kg^−1^.min^−1^	= Oxygen cost of steady-state exercise= Economy, mean power output, or 10-km TT completion time
Whitfield et al. (49)	Young active males (*n =* 10)	Determine if BRJ altered various indices of mitochondrial bioenergetics	−7 day supplement consumption with BRJ (280 ml day^−1^, 26 mmol ${\text{NO}}_{3}^{-}$) -Performed 20 min of cycling (10 min at 50 and 70% VO2peak) 48 h before “Pre” and “Post” 5 day of supplement consumption	↓Oxygen cost↑ H_2_O_2_= Mitochondrial coupling and respiratory efficiency
Betteridge et al. (50)	Healthy recreationally active males (*n =* 8)	Assess the influence of BRJ supplementation on oxygen consumption, glucose kinetics, or skeletal muscle metabolism during submaximal exercise	-BR; 8 mmol ${\text{NO}}_{3}^{-}$ 2.5 h later, participants cycled for 60 min on an ergometer at 65% of VO_2peak_	= Oxygen consumption↑Muscle creatine, lactate, and phosphorylated acetyl CoA carboxylase during exercise
Thompson et al. (51)	Recreationally-active subjects (*n =* 36)	Investigated the independent and combined performance and physiological effects of SIT and ${\text{NO}}_{3}^{-}$ ingestion	-BRJ; ~6.4 mmol of ${\text{NO}}_{3}^{-}$ per 70 ml for 28 days -Subjects consumed 2 × 70 ml of their allocated supplement 2.5 h before the exercise tests	↓ O_2_ cost↑ Peak work rate- SIT and BR ingestion provided greater improvements in incremental exercise performance compared to either intervention alone and led to greater improvements in some indices of muscle metabolic adaptation
Santana et al. (52)	Healthy participants (*n =* 16)	Influence of inorganic ${\text{NO}}_{3}^{-}$ ingestion combined to a short training program on 10-km running TT performance, maximum and average power on a Wingate test, and [La^−^] in recreational runners	-Consumed 750 mg/day (~12 mmol) of ${\text{NO}}_{3}^{-}$ plus 5 g of resistant starch, for 30 days	- Improved 10-km TT performance and kept blood [La^−^] steady
Pawlak-Chaouch et al. (53)	Elite endurance athletes (*n =* 17)	Investigated the effects of BR consumption on enhances the tolerance to SIE	−3day BR supplementation (340 mg/day) -Exercise test: 15-s cycling exercise bouts at 170% of the maximal aerobic power interspersed with 30-s passive recovery period	= Tolerance to SIE= VO_2_ and local muscle O_2_ delivery and extraction
Wickham et al. (54)	Recreational active females (*n =* 12)	Determine the influence of acute and chronic BRJ ingestion on submaximal exercise VO2, TT performance	-Supplementation acutely (2.5 h) and chronically (8 days) with 280 mL BRJ/d (~26 mmoles ${\text{NO}}_{3}^{-}$) -Cycled for 10 min at 50 and 70% VO_2peak_	= MVC, voluntary activation, peak twitch torque, time to peak torque, or half relaxation time.- Not reduce O_2_ cost of submaximal exercise
Behrens et al. (55)	Adults with obesity (body mass index >30 kg/m^2^) (*n =* 16)	Investigate the effect of BRJ on ET, EE, and cardiometabolic health	-${\text{NO}}_{3}^{-}$ rich BRJ (BRJ; ~6.4 mmol of ${\text{NO}}_{3}^{-}$ per 70 ml), 2.5 h before exercise	-Improved exercise efficiency during submaximal exercise by 7%, and time to exhaustion by 15% compared to other conditions
**BR supplementation and antioxidant**				
Roth (56)	Recreationally active (*n =* 30)	Examine the influence of acute versus chronic BR supplement consumption on oxidative stress, and antioxidant capacity (SOD)	- Consuming BR for 7 days, 140 ml or 0.8 g of ${\text{NO}}_{3}^{-}$	↑ Antioxidant capacity (SOD)
Singh et al. (40)	Infantry soldiers (*n =* 30)	Investigated the influence of 15 days dietary ${\text{NO}}_{3}^{-}$ supplement consumption on high density lipoprotein-cholesterol and oxidative Stress	−400 ml BRJ (consumed twice daily) for 15 days	↑ Plasma total antioxidant capacity ↓ Stress markers plasma hydroperoxides
Whitfield et al. (33)	Recreationally active males (*n =* 8)	Investigate the influence of 7 d of BRJ ingestion on skeletal muscle contractile characteristics and function	−7 days of BRJ ingestion (280 mL.d^−1^, 26 mmol ${\text{NO}}_{3}^{-}$) - Performed 20 min of cycling (10 min at 50 and 70% VO_2peak_) 48 h before “Pre” and “Post” 5 day of supplement consumption	= GSH:GSSG ratio
Kozlowska et al. (57)	Elite fencers (*n =* 20)	Examine the effects of diet and active substances in BRJ supplementation on the oxidative stress, inflammation, and muscle damage in elite fencers	- Received freeze-dried BRJ in the amount of 26 g per day, which corresponded to one glass of juice (200 ml), 4 weeks	↑Lipid peroxidation,GPx1 activity↑ VO_2max_ and changes of this parameter were negatively related to changes of LDH serum activity, as well as to the concentrations of β-carotene and MDA.

*=, No significant difference; ↓, significantly decreased responses; ↑, significantly increased responses; BRJ, Beetroot juice; TT, Time trial; VO_2_, Oxygen uptake; ${\text{NO}}_{3}^{-}$, Nitrate, ${\text{NO}}_{2}^{-,}$ Nitrite; MVS, Maximum voluntary strength; EE, Exercise efficiency; O_2_, Oxygen; IO, Individuals with obesity; ET, Exercise tolerance; CF, CrossFit; ROS, Reactive oxygen species; RT, Resistance training; RPE, Rating of perceived exertion; H_2_O_2_, Hydrogen peroxide; POMS, Profile of mood states; NR, Nitrate-Rich; La^−^, Lactate concentration;, SIE, Supramaximal intensity intermittent exercise; CMJ, Countermovement jump; BR, Beetroot; SIT, Sprint interval training; GPx1, Glutathione peroxidase 1; TAC, Total antioxidant capacity; TP, Total polyphenol; SOD, Superoxide dismutase; DNA, Deoxyribonucleic acid; GSH:GSSG, The ratio of reduced/oxidized glutathione; GST, Glutathione S-transferase; NQO1, NAD(P)H:quinone oxidoreductase 1; NP-SH, Non-protein sulfhydryl; GSH, Glutathione; LDH, Lactic acid dehydrogenase; MDA, Malondialdehyde*.

**Figure 3:**
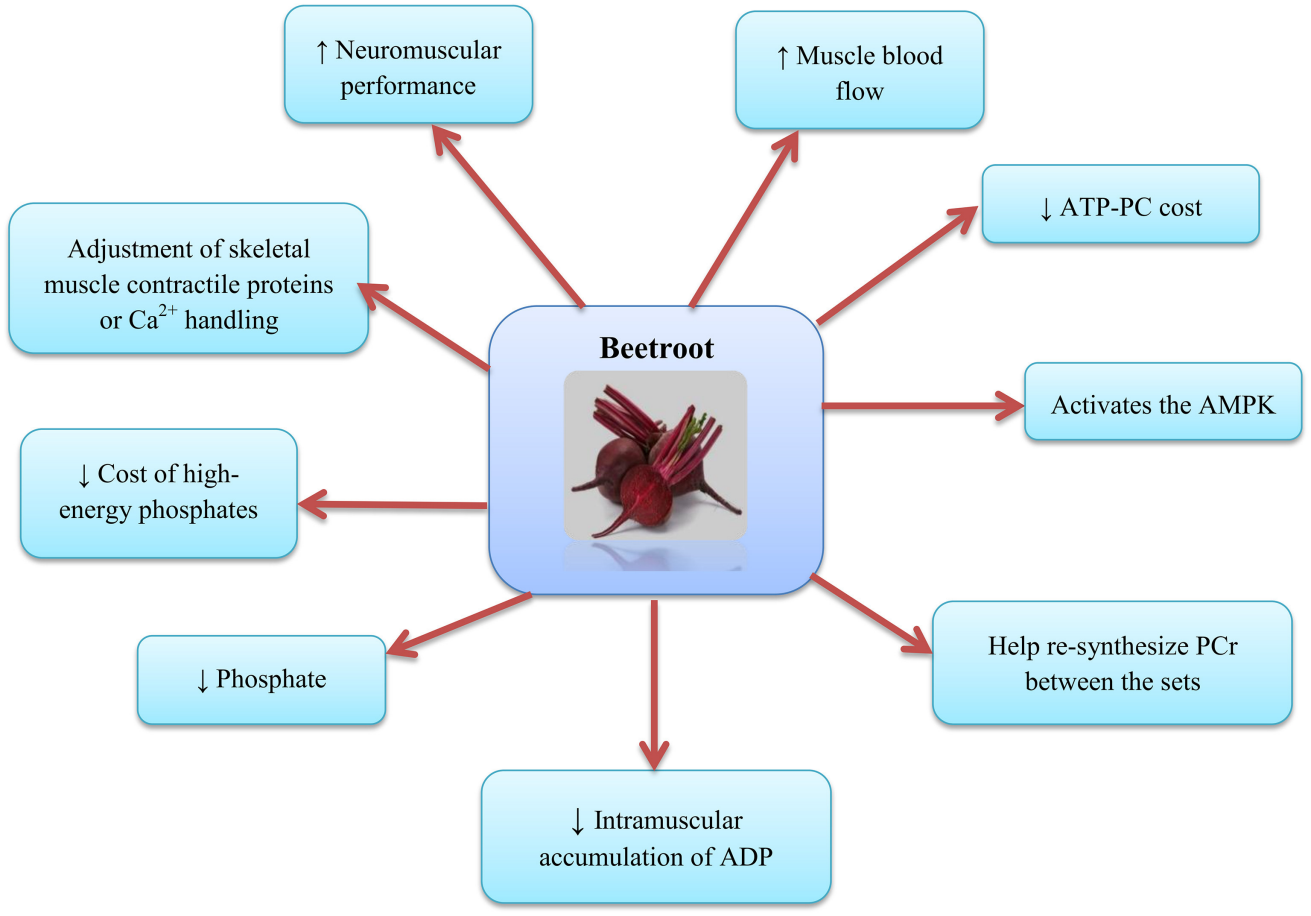
Beetroot supplementation and the skeletal muscle. AMPK, AMP-activated protein kinase; PCr, Phosphocreatine; ADP, Adenosine diphosphate.

The mechanism by which ${\text{NO}}_{3}^{-}$ can enhance contractile performance in skeletal muscle is the adjustment of contractile proteins or Ca^2+^ handling in skeletal muscle after translation (63) (as shown in Figure 3). In fact, NO can respond with protein thiols [e.g., groups containing sulfhydryl groups, protein thiol (RSH) or thiolate anion (RS^−^)] to form nitrosothiols (RSNO) groups in a reversible process called S-nitrosylation (64). S-nitrosylation and denitrosylation alter the composition of the structure and thus the function of proteins (65). For example, NO reaction with heavy chains of myosin S-nitrosylate has been reported in skeletal muscle, causing heightened contractile force (66). Potential effect of S-nitrosylation on the stimulation-contraction pair is complex given that different contraction-related proteins can post translate into the cysteine residues on the thiols such as myosin (67), troponin (68), sarcoendoplasmic reticulum (SR) calcium transport ATPase (SERCA) (69), and ryanodine receptors (RyRs) (70, 71) to undergo irreversible changes; these post-translational protein changes probably depend on the interactions between NO, the reactive oxygen species (ROS), and the bioavailability of glutathione (72). Furthermore, RyR proteins contain a significant quantity of sulfhydryl groups in comparison with the other contractile proteins, and this supports the hypothesis that modulation of RyR and release of ${\text{Ca}}_{2}^{+}$ mediated NO can help increase muscle contraction following the ${\text{NO}}_{3}^{-}$ consumption. The important point is that, these influences can be free of alterations in the substance of ${\text{Ca}}_{2}^{+}$-handling proteins (73). Further potential alterations in the excitation-contraction coupling proteins, ${\text{NO}}_{3}^{-}$ supplement consumption has been proposed to change the high-energy phosphate turnover and phosphorus metabolites in the skeletal muscle (20, 29). ${\text{NO}}_{3}^{-}$ reduces the cost of high-energy phosphates in the generation of skeletal muscle contraction (20, 29) and the intramuscular accumulation of adenosine diphosphate (ADP) and phosphate, factors that are expected to reduce the expansion of fatigue in skeletal muscle (74). Furthermore, ${\text{NO}}_{3}^{-}$ supplementation increases the muscle blood flow (58) and may help re-synthesize phosphocreatine (PCr) between the sets (75, 76) and force recovery and performance (77–79). Molecular mechanisms for the oxidative metabolism in skeletal muscle and hypertrophy adaptations due to exercise are diverse and possibly contradictory (80, 81). For example, it has been reported that ${\text{NO}}_{2}^{-}$ activates the AMP-activated protein kinase (AMPK) (82), while the main regulatory factor is adaptability of the skeletal muscle oxidative metabolism, but interferes with mammalian target of rapamycin complex 1 (mTORC1) signaling, a major regulator of skeletal muscle hypertrophy (83, 84).

Another physiological basis may be due to the effects of certain types of muscle fibers on the ${\text{NO}}_{3}^{-}$ supplement consumption (85). Ferguson et al. stated that the BR consumption elevate blood flow to limbs and skeletal muscle [preferably with fast-twitch fibers (type 2)] (58). Due to the fact that oxygen supply is a limiting factor in the adenosine triphosphate (ATP)-PC regeneration, and lactate clearance perhaps affect the muscle strength generation (86, 87), improving blood flow to type 2 fibers may improve and maintain the muscle strength and ultimately lead to improved performance in the resistance training (28). Moreover, the results of Whitfield et al. showed that BR ingestion enhance force generation at low excitation frequencies and in the human skeletal muscle, this is independent of the change in the redox stress or the expression of protein targets related to calcium operation (33). It has been shown that the amount of ATP turnover in myocyte contraction is largely determined by the activity of actomyosin ATPase and ATPase-${\text{Ca}}_{2}^{+}$ (88). Also, NO causes lagging myosin cycling kinetics and increases force production with each power stroke (66), and decreases activity of the ATPase-${\text{Ca}}_{2}^{+}$ (69). Increased NO production after the BR supplementation may decreases ATP turnover by declining the activity of actomyosin ATPase or ATPase-Ca2^+^. Intracellular accumulation of ADP and Pi and PCr degradation are also blunted following the ${\text{NO}}_{3}^{-}$ supplement consumption (20). Dietary ${\text{NO}}_{3}^{-}$ supplementation appears to improve the link between the ATP hydrolysis and muscle force generation, and this is a significant determinant of oxygen uptake (VO_2_) reduction during the exercise. Alterations in the ADP, Pi, and PCr following the ${\text{NO}}_{3}^{-}$ consumption were predicted to decrease stimuli for the increased oxidative phosphorylation relying to the existing respiratory control models (89–91).

Effective mechanisms for the effects of BR on the performance of resistance training may be the result of increased neuromuscular efficiency (31), decreased ATP-PC cost (92), and alterations in the calcium handling (59). The results of Williams et al. showed that the BR is a sound, natural and efficient ergogenic supplement that has a positive effect on the speed, strength and total repetitions during the chest press exercises with free weights (36). Moreover, the results of research by Flanagan et al. showed that the ${\text{NO}}_{3}^{-}$ supplementation improves neuromuscular efficiency of force generation and its effects become more pronounced with fatigue. They suggested that the observed improvement in the neuromuscular function may be due to the increased sarcoplasmic reticulum Ca^2+^ release (31). Hernandez et al. observed that the increase in the contractile force in fast-twitch muscles due to ${\text{NO}}_{3}^{-}$ supplementation was related to the increase in the tetanic Ca^2+^ concentrations (59). Furthermore, analysis of glucose kinetics after the ${\text{NO}}_{3}^{-}$ intake provides important insights into specifying the impacts of ${\text{NO}}_{3}^{-}$ on glucose metabolism during the physical activity in humans. In addition, quantitative studies have examined the influence of ${\text{NO}}_{3}^{-}$/BR consumption on the AMPK signaling during training (AMPK is the skeletal muscle energy sensor that is activated by training) (93, 94). A study by Betteridge et al. on the effect of BR on the glucose kinetics, muscle metabolism, AMPK signaling and oxygen consumption, showed that the acute BR consumption had no significant effects on the glucose disposal, Acetyl-CoA carboxylase (ACC) phosphorylation, muscle metabolism, oxygen consumption, or RER during the moderate-intensity physical activity in healthy men (50).

In addition to muscle strength, improving muscle endurance during the resistance training can be due to the role of NO, because it participates in the regulation of blood pressure (95) by vasodilatory, along with the capability to prevent blood coagulation (96). Moreover, to the endogenous production of L-arginine oxidation, there is a metabolic oxidation pathway, ${\text{NO}}_{3}^{-}$-${\text{NO}}_{2}^{-}$-NO, which is independent of the NO synthases (NOS) (97). Thus, in exercise that involve main components of muscular endurance, helping the ${\text{NO}}_{3}^{-}$ through BR supplement is a more effective way to improve the NO generation, in favor of greater blood volume and hence oxygen. This becomes even more important when exercise specifically leads to an acidic and hypoxic body environment, a condition in which a decrease in the ${\text{NO}}_{3}^{-}$ metabolism increases the NO precursor supplement activity (97). More oxygen supply causes delay in the onset of muscle fatigue in sportive performance (98) and leads to enhanced energy generation in the form of ATP through the aerobic metabolic pathways. In more intense and longer exercise, delays in the onset of anaerobic metabolic pathways lead to the increased oxygen consumption which is required for ATP production (98). Consequently, the use of ${\text{NO}}_{3}^{-}$ can improve sport performance, because of the vasodilatory function and providing more oxygen to the muscles enhances the maximum oxygen consumption of individuals and decreases the oxygen consumption for exercise. All these reduce the cost of ATP and, therefore, alter intramuscular substrates and metabolic generation (PCr, ADP, Pi), increases muscle oxygenation (12) and delays the onset of muscle fatigue. As a result, decreased oxygen consumption and lower ATP costs cause delay in the lactate generation (98). Since lactate is an indicator of cooperation of the glycolysis in the metabolism, after consuming BR with the same concentrations of blood lactate, increasing the total number of repetitions obtained can indicate energy efficiency (99). Accordingly, Bailey et al. showed that ingestion of BR decreases the cost of oxygen and the rate of PCr degradation during the low and high intensity training without affecting muscle pH and improving training performance (20).

In addition, studies have investigated the influence of BR on the rating of perceived exertion (RPE); some studies have suggested improved performance without a considerable effect on the RPE (32, 100, 101) and other studies have shown a decrease in the RPE values (37, 102). Mosher et al. stated that RPE muscle endurance in resistance training was not affected by 6 days of ${\text{NO}}_{3}^{-}$ consumption (32). In contrast, the results of Jodra et al. showed that the BR consumption can reduce muscle RPE (37). Possible mechanisms that may explain the effect of BR on the RPE include higher blood flow to the brain frontal lobe, which regulates motor control and decision-making, contributing to a subjective perception of effort (103), and possibly increasing athletic performance (37). In addition, it should be noted that the maintenance of RPE after the BR supplement consumption can be the result of decreased central motor command due to the contractile operation maintained during the exercise (102), as RPE displays a central feedback process in which a motor order output is dispatched from the motor zones to the sensory brain to allow conscious awareness of the actions relevant to motor yield (103). During strenuous contractions, a gradual enhance in the RPE may indicate an increment in the central motor command required for training-induced deficiencies in the contractile muscle function to ensure that there is sufficient output power to maintain work (104). In fact, decreased blood flow to the brain during training is known to be a major cause of fatigue (105). Therefore, increased cerebral blood flow can play a role in reducing the muscle RPE and improving function after the BR supplementation (37). Based on studies (Table 2), acute (2–3 h) (28, 35–37, 39), short-term (<15 day) (30–34, 38), and long-term (≥15 day) (29) BR supplementation is effective for building muscle adaptations.

The results of Wylie et al. showed that the skeletal muscle ${\text{NO}}_{3}^{-}$ stores are sensitive to ${\text{NO}}_{3}^{-}$ ingestion and probably help to NO production during training. They reported that skeletal muscle contains sialin (${\text{NO}}_{3}^{-}$ transporter) and xanthine oxidoreductase and stated that skeletal muscle also plays an main role in the transport, storage, and metabolism of the ${\text{NO}}_{3}^{-}$. They also stated that baseline ${\text{NO}}_{3}^{-}$ levels and ${\text{NO}}_{2}^{-}$ concentrations in muscle are much higher than plasma, and ${\text{NO}}_{3}^{-}$ intake increases plasma and muscle ${\text{NO}}_{3}^{-}$ concentrations (106). Also, Srihirun et al. concluded that muscle cells rely on the transport of ${\text{NO}}_{3}^{-}$ from external sources for achieving sufficient ${\text{NO}}_{3}^{-}$ stores in order to convert sufficiently to ${\text{NO}}_{2}^{-}$ and NO, and that muscle cells normally cannot raise endogenous ${\text{NO}}_{3}^{-}$ levels to high levels for supporting all routine and emergency procedures. They also stated that sialin and chloride channel 1 (CLC1) (${\text{NO}}_{3}^{-}$ transporters) play an important role (107). During skeletal muscle contraction, increased the NO storage supports increased mitochondrial respiration, glucose uptake, and other functions (64).

## The Impact of BR Supplementation on the Hormonal Response

NO is an intracellular and extracellular messenger for the adjustment of several cellular functions, such as changes in the hormone secretion (108, 109) for anabolic and catabolic aim. It is one of the major mediators of stress involved in the acute hypothalamic-pituitary-adrenal (HPA) response to training. The effects of NO on the pituitary and adrenal cortex have been confirmed (110) and it has been suggested that the cortisol secretion can be stimulated directly by the NO concentration after tadalafil administration (111). Activation of the HPA axis during the training greatly increases the cortisol levels (112), which acts as a metabolic and catabolic hormone (113) by mobilizing the glucose (114), endogenous stores of amino acids (115), and fatty acids (116), enhances the availability of all fuel substrates; as a result, BR improves performance (42).

In addition, NO plays the main role in anabolic hormones; Valenti et al. reported that the NO has a biphasic effect on testosterone secretion, NO inhibits testosterone secretion at higher levels and stimulant at low concentrations; the stimulant effect of NO is mediated by cGMP (117). Like NO, testosterone perhaps stimulates the vasodilator effect (118, 119). However, testosterone appears to cause vasodilatation at concentrations above 10 μmol/L, but at low physiological concentrations, NO appears to be involved in the vasodilatory effects of this hormone (120). It appears that different concentrations of NO can alter the hormonal response of cortisol and testosterone (42). Garnacho-Castaño et al. concluded that cortisol and testosterone levels were markedly increased in the BR juice and placebo intake groups. They stated that most alterations observed in the cortisol levels after the BR consumption maybe associated with the ${\text{NO}}_{3}^{-}$-${\text{NO}}_{2}^{-}$-NO pathway and more studies are needed to confirm this hypothesis (42). Also, the results of Roberts et al. Show an increase in growth hormone (GH) concentration in rats and humans due to ${\text{NO}}_{3}^{-}$ consumption. They state that peroxisome proliferator–activated receptor gamma coactivator-1 (PGC-1) and NO, by secreting muscle gamma-aminobutyric acid (GABA) and peripheral GABA concentrations, may help release exercise-stimulated GH (41).

## The Influence of BR Supplementation on the Nervous Function

The NO is produced endogenously in a variety of cells In a mammalian organism, such as nerve cells, endothelial cells, and macrophages, by a category of three isozymes called NOS, and uses L-arginine as a substrate (121). Endogenously synthesized NO has been shown to not only act as an intercellular messenger, but also to spread rapidly and affect NO target cells. Thus, the released NO may affect nerve cells over a wide area (122). The NO is a messenger molecule with numerous molecular targets among other servo-regulatory control functions including neurotransmission (123). Considering the modulation of nerve cell function by the NO *in vivo* and *in vitro* studies, it has been shown that in all brain structures, NO modulates the release of several neurotransmitters. Regarding *in vitro* and *in vivo*, NO donors enhance the release of noradrenaline from the hippocampus (124). In the medial preoptic area (125) and the striatum (126), serotonin release is increased by the L-arginine and NO donors, respectively. NO donors have been shown to increase serotonin secretion in the hypothalamus and in the locus coeruleus. In addition, NO has a moderating role; high levels of NO enhance serotonin values in the hypothalamus, while slight concentrations of NO appear to decrease it (127). Increased serotonin modulates fatigue in long-term activity (128). Serotonin has metabolic and developmental effects on the skeletal and cardiac muscles; it regulates energy balance and glucose uptake by the skeletal muscle and prevents insulin resistance (129). In a study conducted by Kozlowska et al. significant changes in the signal intensity induced by the tyrosine and tryptophan metabolites were observed, especially from the noradrenaline, adrenaline pathways, and serotonin metabolism as well as lipid peroxidation (44).

In the pathway of tyrosine metabolism, most of the experimental metabolites identified come from the adrenaline and noradrenaline degradation subpathway. L-tyrozine is changed to L-dopa and further to dopamine with pyridoxal phosphate as a cofactor (130). Dopamine may modulate skeletal muscle activity (131) and affect mitochondrial activity. Improper regulation of the dopamine stimulates oxidative mechanisms (132). Moreover, dopamine contributes in the synthesis of noradrenaline and adrenaline as a precursor (130). In nerve cells that use the dopamine as a transmitter, no enzymatic changes occur, but neurons applying noradrenaline as a transmitter, contain other enzymes that convert dopamine to noradrenaline and oxygen, also ascorbic acid is the cofactor of this process. Neurons that use adrenaline as a transmitter contain an another enzyme that catalyzes the conversion of noradrenaline to adrenaline (133).

**Figure 4:**
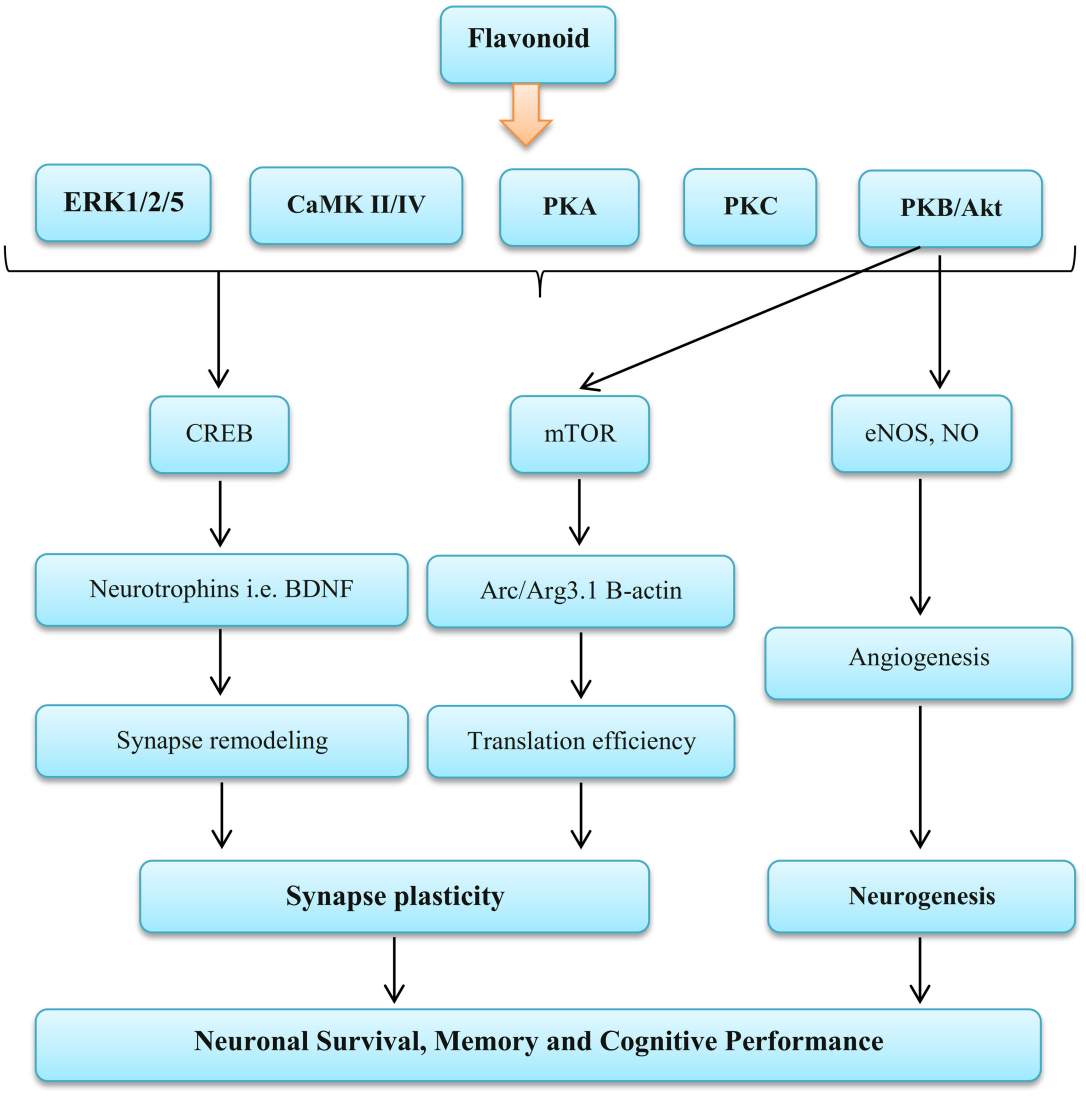
Influence Flavonoid on the nervous function. ERK, Extracellular signal-regulated kinases; CaMK II/IV, Calmodulin-dependent protein kinase II/IV; PKA, Protein kinase A; PKC, Protein kinase C; PKB/AKT, Protein kinase B; CREB, cAMP-response element binding protein; mTOR, Mammalian target of rapamycin; eNOS, Endothelial nitric oxide systems; NO, Nitric oxide; BDNF, Brain-derived neurotrophic factor; Arc/Arg3.1, Activity-regulated cytoskeleton-associated protein. Source: Vauzour et al. (142).

Studies have reported that the brain stem NO bioavailability maybe inhibit sympathetic nerve activity (134–136). In addition, it has been shown that the NO derived from both endothelial NO synthase and neuronal NO synthase inhibits sympathetic vasoconstriction in human and animal specimens (37, 102, 137). Reduction muscle sympathetic activity causes an increase in vasodilation and skeletal muscle blood flow (138). Enhance vasodilation is an important factor in regulating cardiac output, and this is largely related to oxygen delivery (139). In general, evidence has shown that inhibition of the NO generation prevents inhibition of sympathetic vasoconstriction in the skeletal muscle in contracted or rested forms in humans and rodents (103). In contrast, findings of de Vries and DeLorey showed that increasing the NO bioavailability through acute (2.5 h) the BR supplement consumption does not change the sympathetic vasoconstrictor responsiveness to sympathoexcitation at rest or during training (43). In addition, other components of BR can affect nerve function, including flavonoids. Flavonoids exert complex functions on NO synthesis and bioavailability that may increase or decrease NO levels (140). Flavonoids activate the extracellular signal-regulated kinase (ERK)—cAMP response element binding protein (CREB) pathway and the phosphoinositide 3 (PI3) kinase mTOR cascade, leading to changes in synaptic flexibility. Also they are able to affect neurogenesis by activating PI3 kinase—protein kinase B (Akt)—endothelial NOS (eNOS) (141, 142) (as shown in Figure 4).

## The Influence of BR Supplementation on the O_2_ Cost and Mitochondria

Dietary ${\text{NO}}_{3}^{-}$, which is studied as a supplement to the BR, can increase adaptation to endurance exercise due to its ability to enhance ${\text{NO}}_{2}^{-}$ and NO in plasma (15). By increasing mitochondrial efficiency (22), decreasing the cost of oxygen to muscle contraction (20) and enhancement the contractile force in the fast-twitch muscles (111), it can increase the intensity of exercise (therefore the total work done in a training session) (143). Increased mitochondrial biogenesis (stimulates PGC-1 activation through stimulation of NO by guanylate cyclase) (144) and changes the muscle fiber type to a more oxidative phenotype when combined with the SIT [through the role of nuclear factor activated T cells (NFAT)—calcineurin] (51, 145–147). This is possibly related to the increased mitochondrial hydrogen peroxide (49).

Last evidence indicates that the dietary ${\text{NO}}_{3}^{-}$ supplement consumption perhaps decreases the cost of O_2_ exercise by reducing the cost of ATP skeletal muscle contraction (20, 33, 59, 148). The major costs of ATP during the skeletal muscle contraction are via myosin ATPase and SERCA (88). One of the main regulatory impacts of the NO is its capability to competitively and reversibly bind to *c* oxidase (COX) and therefore prevent mitochondrial respiration. This was first shown by adding NO donors to the isolated mitochondria (149, 150) and later by showing that NOS inhibition was associated with enhancement of oxygen consumption in the resting muscles of dogs and other organs (151, 152). Even if it is shown that the NO prevents COX, this can automatically lead to a decrease in the mitochondrial respiration. In human mitochondria, COX has a capacity of more than 8 times the maximum flux of the ETS (electron transport system). Therefore, respiration may not be affected to the extent that the COX limits speed. Although the causes for this extra capacity are unclear, it has been proposed that it may be needful to stop intense inhibition of the COX by NO under natural physiological conditions. Additional COX capacity is significant to maintain a sufficient increase in oxygen affinity to the mitochondria (153).

COX must be severely inhibited before reducing oxygen consumption throughout the body, because peripheral mitochondrial respiratory capacity exceeds systemic oxygen delivery. Actually, even when isolated mitochondria are motivated by the ADP for full respiration at saturating oxygen tension, only part of the total COX capacity is utilized (154). It has been shown that when endogenous NO synthesis is blocked, the body's oxygen consumption increases frequently. This indicates that the physiological level of NO around 20 nM actually has an impact on the tissue oxygen consumption (151, 152). Studies have shown that NO indirectly improves oxidative phosphorylation efficiency by inhibiting COX (155). COX transfers electrons to oxygen and eventually water is formed, and, at the same time, protons are pumped through the inner membrane of the mitochondria. Although, electrons can move via the COX protein free of pumping protons (156). At the time of COX inhibition by the respiration and the NO is somewhat diminished, documents show that ATP production is sustained to a higher degree compared to the oxygen utilization, resulting in an elevation in the amount of ATP produced per oxygen consumption (P/O ratio) (155). This is supported by the fact that inhibition of endogenous NO generation enhances oxygen consumption without altering ATP production (157). Binding of the NO to the COX can also cause intracellular signaling events, including oxygen diversion to non-respiratory substrates and ROS production with potentially destructive effects (158). These NO-elicited events operate as triggers by which mitochondria regulate signal transduction cascades engaged in the excitation of defense mechanisms and adaptive responses in cells (159).

One of the main components of BR is flavonoids. Flavonoids can affect mitochondrial Ca^2+^ uniporter within the cell and increase mitochondrial Ca^2+^ concentrations. Ca^2+^ regulates various pathways, including the pathway that stimulates eNOS, helps increase NO production, stimulates the flow of K^2+^ and Ca^2+^, and increases the polarization of cell membranes in endothelial cells (160). These cause the muscles in the blood vessels to relax, thereby lowering blood pressure and enhancement blood flow (17, 161). Also, an increase in mitochondrial Ca^2+^ may by upregulation pyruvate dehydrogenase and increasing the potential of the mitochondrial membrane, stimulate oxidative metabolism; increasing the concentration of ATP produced reduces oxidative stress and muscle damage, and ultimately increases productivity power (162).

Results of Santana et al. (52) and Wylie et al. (163) showed that the ${\text{NO}}_{3}^{-}$ can transfer energy source from anaerobic to oxidative sources. They stated that the benefits of ${\text{NO}}_{3}^{-}$ consumption on the concentration of blood lactate in endurance running or strenuous exercise depended on the period of supplementation, and the level of physical fitness of the subjects. In addition, the results of Papadopoulos et al. (35) and Whitfield et al. (49) showed that the mechanical basis for reducing VO_2_ of the whole body after receiving ${\text{NO}}_{3}^{-}$ in the diet does not seem to be an increase in the oxidative efficiency of the muscle or mitochondria, but improving some other mechanisms is inherent; for example, improved contractile performance (59), which is associated with the reduced ATP contraction costs (20). In contrast, the results of Pawlak-Chaouch et al. stated that 3 day the BR supplementation had no effect on the VO_2_ and local muscle O_2_ delivery and extraction in elite endurance athletes (53). Moreover, Wickham et al. showed that acute (2.5 h) and chronic (8 day) BR consumption has no ergogenically effect among the active women and does not improve aerobic function (54). Furthermore, Arnold et al. concluded that the acute (2.5 h) ${\text{NO}}_{3}^{-}$ ingestion did not increase the performance of endurance runners in normobaric hypoxia. They stated that the dose used in this study was not sufficient to produce positive effects. The effects of ${\text{NO}}_{3}^{-}$ consumption on the athletic performance in hypoxia depend on the duration, and intensity of exercise (47). According to research (Table 2), acute (2.5–3 h) BR supplementation (18, 48–50, 55, 56) does not seem to have much effect on the O_2_ Cost and mitochondria, and seems short-term (<15 day) (45, 46, 51, 54, 55) and long-term (≥15 day) (51, 52) BR supplementation to have a greater effect. The level of exercise/fitness of the subjects are a very important factor in the effect of the BR supplementation (21, 164). In endurance-trained subjects, especially elite endurance athletes, fewer adaptations occur as a result of the BR supplementation (21). The BR supplementation is less effective in people who are higher fitness level (164). Considering the fitness level of the subjects, it can be said that acute supplementation (2.5–3 h) in trained people probably has no effect (47, 48, 50) and requires longer supplementation courses.

## Antioxidant Effects of the BR Supplementation

**Figure 5:**
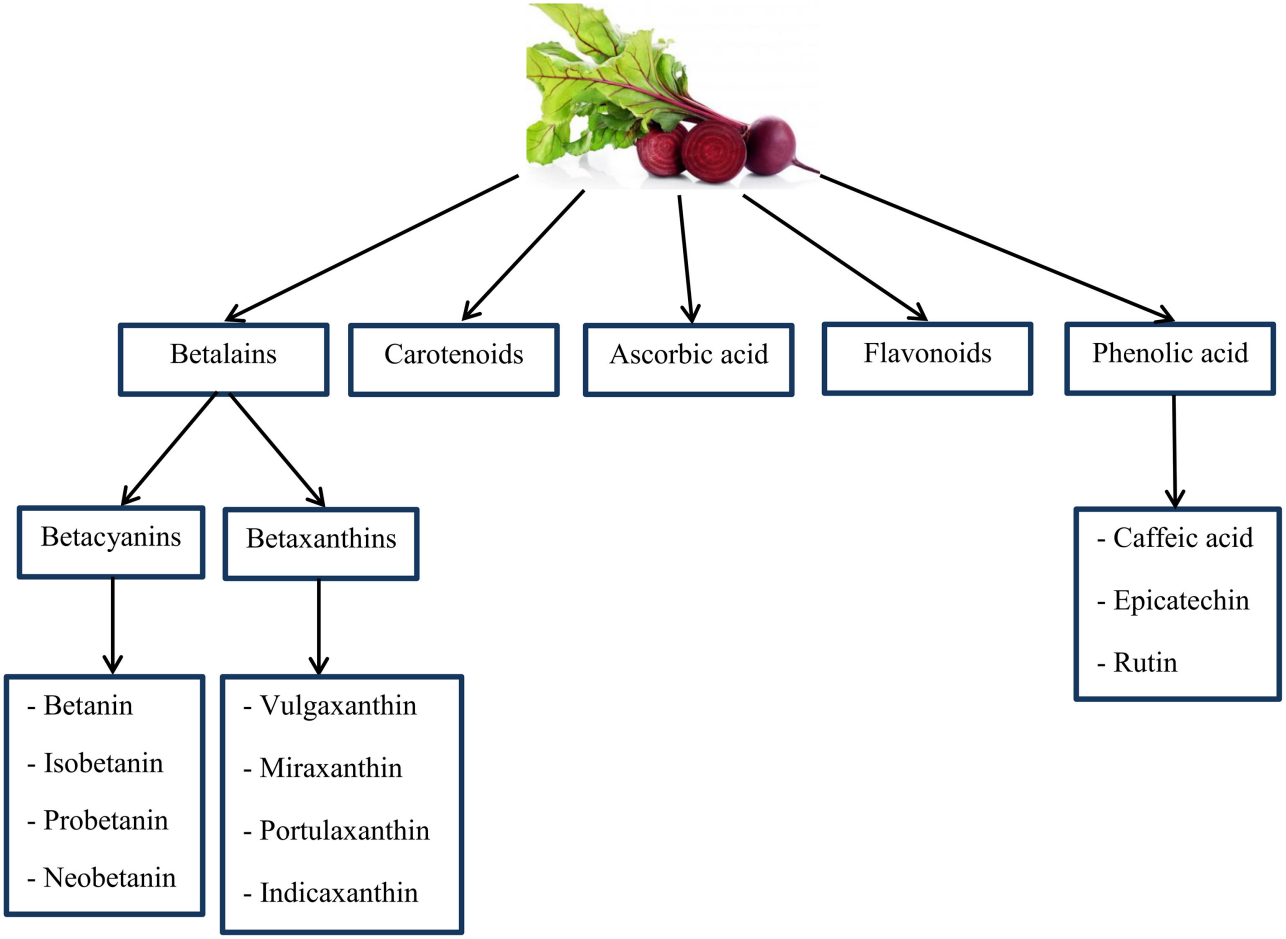
An overview of important chemical compounds in Beetroot that may have antioxidant properties. Source: Georgiev et al. (165); Kujala et al. (166); Wootton-Beard and Ryan (167); Lee et al. (168); Vulic et al. (169).

BR is the main source of chemical compounds including: ascorbic acid, carotenoids, phenolic acid, flavonoids (165–167), and betalains (a group of bioactive pigments) (168, 169). Many animal models have displayed that betalains contain vast anti-inflammatory and antioxidant features properties (169–173). In addition, highly active phenolics include caffeic acid, epicatechin, and rutin are known as the organic antioxidants (165, 174) (as shown in Figure 5). Also, the BR is a dense origin of inorganic ${\text{NO}}_{3}^{-}$, as a substrate for the NO synthesis (175). Some studies have reported that ${\text{NO}}_{2}^{-}$ and NO to inhibit radical stablishment and repel ROS and RNS; this is indicating the antioxidant effects of ${\text{NO}}_{3}^{-}$ (176, 177).

Betalains and betacyanins (betanin and isobetanin) are complementary components of the BR juice that protect against DNA, protein and lipid damage (178, 179). Physical exercise releases oxidative stress and BR phytochemicals prevent the formation of radicals (2,2-diphenyl-1-picrylhydrazyl and 3-ethylbenzothiazoline-6-sulfonicacid) (180). According to research, administration of the ${\text{NO}}_{3}^{-}$ at a rate of 8 ml per kg of body weight per day reduces protein oxidation, DNA damage and lipid peroxidation in rats (178). Wootton-Beard and Ryan recognized the increment in simulated digestion, by the consumption of BR juice as a function of antioxidant and also affirm the juice components are phytochemicals which show similar functions and their structure are changed (167). BR supplement provides a significant source of dietary polyphenols and due to the many health benefits of polyphenols, supplements containing large amounts of it can be beneficial to individuals (167). However, the BR shows possible antioxidant properties via scavenging mechanism regarding radical species. Phytochemicals of BR through signaling pathways inhibit inflammatory diseases (181).

Among the betalains, betanin (betanidin 5-O-b-D-glucoside) is a major phytochemical representative, a water-soluble nitrogen heterocyclic compound that gives the BR a red-violet color (182). In addition, the betanin could inhibit low-density lipoprotein (LDL) and lipid membranes peroxidation because of its bioactive composition, modulating ROS production and gene expression to reduce the inflammatory release of cytokines and increase antioxidant enzyme activities (183). Some biological effects shown by betanin are reduced in two redox-sensitive pathways, nuclear factor kappa B (NFkB) and nuclear factor erythroid-2-related factor (Nrf2)—antioxidant response element (ARE) are the major transcription genes for inflammatory responses, and are detoxifying/antioxidant (184, 185). The betanin resists gastrointestinal digestion, is absorbed by intestinal mucosal epithelial cells, and actively reaches plasma. With the help of hydrogen or electrons, betanin preserves lipid structures and LDL particles, while inducing the transcription of antioxidant genes through the Nrf2 and also simultaneously suppressing pre-inflammatory NFkB pathways, has the ability to scavenger free radicals (186). Also, flavonoids are able to scavenge free radicals directly by donating hydrogen atoms, a capacity that depends on the total number of hydroxyl groups, the pattern of substitution, and the order of the functional groups in terms of nuclear structure (187). The presence of a catechol group in the B-ring, due to its ability to donate hydrogen, increases the formation of a relatively stable flavonoid-derived radical, which is the most important determinant of reactive oxygen and nitrogen species (RONS) inhibition (188). Flavonoids are not only able to activate enzymatic defense, also can regulate the oxidative status of cells by inhibiting oxidative enzymes responsible for the generation of superoxide (include: xanthine oxidase and protein K) (189). Has been shown that flavonoids inhibit nicotinamide adenine dinucleotide phosphate (NADPH) oxidase, cyclooxygenase, lipoxygenase, and microsomal succinoxidase (190).

Kozlowska et al. stated that the chronic BR consumption enhances lipid peroxidation, and improvement of VO_2max_ after the BR supplement consumption seems to be dependent on baseline lactic acid dehydrogenase (LDH) activity, and also, on the serum concentrations of malondialdehyde (MDA) and β-carotene. It is suggested that alterations in the ergogenic effect of BR are negatively dependent to alter in serum concentration of MDA, β-carotene, and the activity of LDH (57). Singh et al. stated that the BR ingestion significantly declined the stress markers of plasma hydroperoxides and improves the antioxidant status (40). Furthermore, Vidal et al. showed a 32% decrease in the function of inflammatory enzymes lipoxygenase and cyclooxygenase by the phenethylamine—betaxanthin and detalan (173) (Table 2). The BR compounds can (antioxidant phytochemicals, iron, vitamin A and vitamin B6) protect the liver against the oxidative stress and inflammation and increase regular detoxification activity (173). It not only prevents oxidative stress, but also reduces anemia. The BR is rich in iron and effectively improves tissue oxygenation and reduces the symptoms of anemia (191). Moreover, with its antioxidant and low fructose properties and high sucrose content, it plays an important role in the diet of athletes (191).

Studies showed that dietary ${\text{NO}}_{3}^{-}$ exerts protective effects on the kidneys, heart and arteries by increasing the bioavailability of NO and reducing the oxidative stress (192, 193). ${\text{NO}}_{3}^{-}$ significantly reduces the production of NADPH-dependent O_2_ in the kidney. It seems that the protective effects of ${\text{NO}}_{3}^{-}$ and its antioxidant properties may be due in part to a decrease in NADPH oxidase activity and ${\text{O}}_{2}^{-}$ production (194). Additional NO signaling mechanisms include the production of other bioactive nitrogen mediators through nitrosation and nitration of proteins and lipids, leading to changes in the protein function and fat signaling (195, 196). Although extra generation of NO and superoxide anions has toxic influences via the formation of peroxynitrite, NO itself can operate as an antioxidant by repressing other reactive radicals (195, 196).

## Conclusion

Overall, evidence suggests that the ${\text{NO}}_{3}^{-}$ supplementation [acute (2–3 h), short-term (<15 day) and long-term (≥15 day)] can modulate contractile function by modulating Ca^2+^ handling and contractile proteins, improve skeletal muscle metabolic control by reducing the cost of high-energy phosphates in the contraction and accumulation of fatigue-related metabolites, and improve skeletal muscle perfusion and may increase the performance of resistance training. It may also improve and maintain muscle productivity strength by improving blood flow to the type 2 fibers, and ultimately improve the performance of resistance training. BR due to its ability to increase ${\text{NO}}_{2}^{-}$ and NO in plasma can increase adaptation to endurance training. By increasing mitochondrial function, decreasing the cost of muscle contraction oxygen, and increasing the contractile force in the fast-twitch muscles, it can increase the intensity of exercise (short-term (<15 day) and long-term (≥15 day) BR supplementation to be more effective). The amount of BR used and the time of its consumption is one of the important and influential factors on its effects on the athletic performance. BR also affects anabolic hormones through NO. It seems that different concentrations of NO can alter the hormonal response of cortisol and testosterone. NO is an intracellular and extracellular messenger for regulating certain cellular functions, including changes in the hormone secretion for the anabolic and catabolic purposes. Few researches have been done in this field (Table 2) and more research are needed for more accurate results.

In addition, the BR is the main source of compounds including: ascorbic acid, carotenoids, phenolic acid and flavonoids, and betalains. The betalains have high antioxidant and anti-inflammatory properties. In addition, highly active phenolics such as caffeic acid, epicatechin, and rutin are known as the organic antioxidants and protect against DNA, protein, and lipid damage. In addition, in terms of RPE, the BR can play a role in reducing muscle RPE and improving function by increasing cerebral blood flow, but the results of research in this regard have been contradictory. Regarding the BR neuronal function, the role of NO can be mentioned, which has numerous molecular purposes among other servo-regulatory control functions, including neurotransmission. In all brain structures, NO appears to regulate the release of several neurotransmitters; NO donors increase the release of noradrenaline from the hippocampus. Further studies are needed to draw more accurate conclusions in this regard.

## Author Contributions

HA designed the review. HA and EE wrote the review. Both authors read and approved the final manuscript.

## Conflict of Interest

The authors declare that the research was conducted in the absence of any commercial or financial relationships that could be construed as a potential conflict of interest.

## References

[1] Ormsbee MJ Lox J Arciero PJ. Beetroot juice and exercise performance. Nutr Diet Suppl. (2013) 5:27–35. 10.2147/NDS.S52664

[2] Olsson H Al-Saadi J Oehler D Pergolizzi J Jr Magnusson P. Physiological effects of beetroot in athletes and patients. Cureus. (2019) 11:e6355. 10.7759/cureus.6355 31938641 PMC6952046

[3] Peeling P Castell LM Derave W de Hon O Burke LM. Sports foods and dietary supplements for optimal function and performance enhancement in track-and-field athletes. Int J Sport Nutr Exerc Metab. (2019) 29:198–209. 10.1123/ijsnem.2018-0271 30299192

[4] Baiao D Silva D Mere Del Aguila E Paschoalin V. Nutritional, bioactive and physicochemical characteristics of different beetroot formulations. Food Additives. (2017) 6. 10.5772/intechopen.69301

[5] Chawla H Parle M Sharma K Yadav M. Beetroot: a health promoting functional food. Inventi Rapid. (2016) 1: 976–3872.

[6] Chhikara N Kushwaha K Sharma P Gat Y Panghal A. Bioactive compounds of beetroot and utilization in food processing industry: a critical review. Food Chem. (2018) 30:192–200. 10.1016/j.foodchem.2018.08.022 30309532

[7] Kale RG Sawate AR Kshirsagar RB Patil BM Mane R. Studies on evaluation of physical and chemical composition of beetroot (Beta vulgaris L.). Int J Chem Stud. (2018) 6:2977–9.

[8] United States Department of Agriculture Agricultural Research Service. Food Data Central: Food and Nutrient Database for Dietary Studies with Standard Reference Legacy. (2019). Available online at: http://www.fdc.nal.usda.gov (accessed March 10, 2020).

[9] Hord NG Tang Y Bryan NS. Food sources of nitrates and nitrites: the physiologic context for potential health benefits. Am J Clin Nutr. (2009) 90:1–10. 10.3945/ajcn.2008.27131 19439460

[10] Lundberg JO Weitzberg E Gladwin MT. The nitrate-nitrite-nitric oxide pathway in physiology and therapeutics. Nat Rev Drug Discov. (2008) 7:156–67. 10.1038/nrd2466 18167491

[11] Weitzberg E Lundberg JO. Novel aspects of dietary nitrate and human health. Annu Rev Nutr. (2013) 33:129–59. 10.1146/annurev-nutr-071812-161159 23642194

[12] Jones AM. Dietary nitrate supplementation and exercise performance. Sports Med. (2014) 44:35–45. 10.1007/s40279-014-0149-y PMC4008816 24791915

[13] Clements W Lee SR Bloomer R. Nitrate ingestion: a review of the health and physical performance effects. Nutrients. (2014) 6:5224–64. 10.3390/nu6115224 25412154 PMC4245587

[14] Govoni M Jansson EA Weitzberg E Lundberg JO. The increase in plasma nitrite after a dietary nitrate load is markedly attenuated by an antibacterial mouthwash. Nitric Oxide. (2008) 19:333–7. 10.1016/j.niox.2008.08.003 18793740

[15] Lundberg JO Govoni M. Inorganic nitrate is a possible source for systemic generation of nitric oxide. Free Radic Biol Med. (2004) 37:395–400. 10.1016/j.freeradbiomed.2004.04.027 15223073

[16] Wylie LJ Kelly J Bailey SJ Blackwell JR Skiba PF Winyard PG. Beetroot juice and exercise: pharmacodynamic and dose-response relationships. J Appl Physiol. (2013) 115:325–36. 10.1152/japplphysiol.00372.2013 23640589

[17] Wylie LJ Mohr M Krustrup P Jackman SR Ermdis G Kelly J. Dietary nitrate supplementation improves team sport-specific intense intermittent exercise performance. Eur J Appl Physiol. (2013) 113:1673–84. 10.1007/s00421-013-2589-8 23370859

[18] Muggeridge DJ Howe CCF Spendiff O Pedlar C James PE Easton C. A single dose of beetroot juice enhances cycling performance in simulated altitude. Med Sci Sports Exerc. (2014) 46:143–50. 10.1249/MSS.0b013e3182a1dc51 23846159

[19] Thompson KG Turner L Prichard J Dodd F Kennedy DO Haskell C. Influence of dietary nitrate supplementation on physiological and cognitive responses to incremental cycle exercise. Respir Physiol Neurobiol. (2014) 193:11–20. 10.1016/j.resp.2013.12.015 24389270

[20] Bailey SJ Fulford J Vanhatalo A Winyard PG Blackwell JR DiMenna FJ. Dietary nitrate supplementation enhances muscle contractile efficiency during knee-extensor exercise in humans. J Appl Physiol. (2010) 109:135–48. 10.1152/japplphysiol.00046.2010 20466802

[21] James PE Willis GR Allen JD Winyard PG Jones AM. Nitrate pharmacokinetics: Taking note of the difference. Nitric Oxide. (2015) 48:44–50. 10.1016/j.niox.2015.04.006 25937621

[22] Larsen FJ Schiffer TA Borniquel S Sahlin K Ekblom B Lundberg JO. Dietary inorganic nitrate improves mitochondrial efficiency in humans. Cell Metab. (2011) 13:149–59. 10.1016/j.cmet.2011.01.004 21284982

[23] Webb AJ Patel N Loukogeorgakis S Okorie M Aboud Z Misra S. Acute blood pressure lowering, vasoprotective, and antiplatelet properties of dietary nitrate via bioconversion to nitrite. Hypertension. (2008) 51:784–90. 10.1161/HYPERTENSIONAHA.107.103523 18250365 PMC2839282

[24] Maiorana A O'Driscoll G Taylor R Green D. Exercise and the nitric oxide vasodilator system. Sports Med. (2003) 33:1013–35. 10.2165/00007256-200333140-00001 14599231

[25] Hickner RC Fisher JS Ehsani AA Kohrt WM. Role of nitric oxide in skeletal muscle blood flow at rest and during dynamic exercise in humans. Am J Physiol. (1997) 273:405–10. 10.1152/ajpheart.1997.273.1.H405 9249515

[26] Gilligan DM Panza JA Kilcoyne CM Waclawiw MA Casino PR Quyyumi AA. Contribution of endothelium-derived nitric oxide to exercise-induced vasodilation. Circulation. (1994) 90:2853–8. 10.1161/01.CIR.90.6.2853 7994830

[27] Maughan RJ Burke LM Dvorak J Larson-Meyer DE Peeling P Phillips SM. IOC consensus statement: dietary supplements and the high-performance athlete. Int J Sport Nutr Exerc Metab. (2018) 28:104–25. 10.1123/ijsnem.2018-0020 29589768

[28] Ranchal-Sanchez A Diaz-Bernier VM La Florida-Villagran D Alonso C Llorente-Cantarero FJ Campos-Perez J. Acute effects of beetroot juice supplements on resistance training: a randomized double-blind crossover. Nutrients. (2020) 12:1912. 10.3390/nu12071912 32605284 PMC7401280

[29] Fulford J Winyard PG Vanhatalo A Bailey SJ Blackwell JR Jones AM. Influence of dietary nitrate supplementation on human skeletal muscle metabolism and force production during maximum voluntary contractions. Pflüg Arch. (2013) 465:517–28. 10.1007/s00424-013-1220-5 23354414

[30] Hoon MW Fornusek C Chapman PG Johnson NA. The effect of nitrate supplementation on muscle contraction in healthy adults. Eur J Sport Sci. (2015) 15:712–9. 10.1080/17461391.2015.1053418 26681629

[31] Flanagan SD Looney DP Miller MJ DuPont WH Pryor L Creighton BC. The effects of nitrate-rich supplementation on neuromuscular efficiency during heavy resistance exercise. J Am Coll Nutr. (2016) 35:100–7. 10.1080/07315724.2015.1081572 26885762

[32] Mosher SL Sparks SA Williams EL Bentley DJ Mc Naughton LR. Ingestion of a nitric oxide enhancing supplement improves resistance exercise performance. J Strength Cond Res. (2016) 30:3520–4. 10.1519/JSC.0000000000001437 27050244

[33] Whitfield J Gamu D Heigenhauser GJ Van Loon LJ Spriet LL Tupling AR. Beetroot juice increases human muscle force without changing Ca2+-handling proteins. Med Sci Sports Exerc. (2017) 49:2016–24. 10.1249/MSS.0000000000001321 28509762

[34] de Oliveira GV Nascimento LADD Volino-Souza M Mesquita JDS Alvares TS. Beetroot-based gel supplementation improves handgrip strength and forearm muscle O_2_ saturation but not exercise tolerance and blood volume in jiu-jitsu athletes. Appl Physiol Nutr Metab. (2018) 43:920–7. 10.1139/apnm-2017-0828 29566543

[35] Papadopoulos S Dipla K Triantafyllou A Nikolaidis MG Kyparos A Touplikioti P. Beetroot increases muscle performance and oxygenation during sustained isometric exercise, but does not alter muscle oxidative efficiency and microvascular reactivity at rest. J Am Coll Nutr. (2018) 37:361–72. 10.1080/07315724.2017.1401497 29425473

[36] Williams TD Martin MP Mintz JA Rogers RR Ballmann CG. Effect of acute beetroot juice supplementation on bench press power, velocity, and repetition volume. J Strength Cond Res. (2020) 34:924–8. 10.1519/JSC.0000000000003509 31913252

[37] Jodra P Domínguez R Sánchez-Oliver AJ Veiga-Herreros P Bailey SJ. Effect of beetroot juice supplementation on mood, perceived exertion, and performance during a 30-s wingate test. Int J Sports Physiol Perform. (2020) 15:243–248. 10.1123/ijspp.2019-0149 31172827

[38] Jonvik KL Hoogervorst D Peelen HB De Niet M Verdijk LB Van Loon LJ. The impact of beetroot juice supplementation on muscular endurance, maximal strength and countermovement jump performance. Eur J Sport Sci. (2020) 20:1–8. 10.1080/17461391.2020.1788649 32594854

[39] Rodríguez-Fernández A Castillo D Raya-González J Domínguez R Bailey SJ. Beetroot juice supplementation increases concentric and eccentric muscle power output. original investigation. J Sci Med Sport. (2020) 24:80–4. 10.1016/j.jsams.2020.05.018 32507624

[40] Singh A Verma S Singh V Nanjappa C Roopa N Raju PS. Beetroot juice supplementation increases high density lipoprotein-cholesterol and reduces oxidative stress in physically active individuals. J Pharm Nut Sci. (2015) 5:179–85. 10.6000/1927-5951.2015.05.03.2

[41] Roberts LD Ashmore T McNally BD Murfitt SA Fernandez BO Feelisch M. Inorganic nitrate mimics exercise-stimulated muscular fiber-type switching and myokine and γ-aminobutyric acid release. Diabetes. (2017) 66:674–88. 10.2337/db16-0843 28028076

[42] Garnacho-Castano MV Palau-Salva G Serra-Paya N Ruiz-Hermosel M Berbell M Vinals X. Understanding the effects of beetroot juice intake on CrossFit performance by assessing hormonal, metabolic and mechanical response: a randomized, double-blind, crossover design. J Int Soc Sports Nutr. (2020) 17:1–12. 10.1186/s12970-020-00388-z 33187518 PMC7666517

[43] de Vries CJ DeLorey DS. Effect of acute dietary nitrate supplementation on sympathetic vasoconstriction at rest and during exercise. J Appl Physiol. (2019) 127:81–8. 10.1152/japplphysiol.01053.2018 31095461 PMC6692739

[44] Kozlowska L Mizera O Mroz A. An untargeted metabolomics approach to investigate the metabolic effect of beetroot juice supplementation in fencers—a preliminary study. Metabolites. (2020) 10:100. 10.3390/metabo10030100 32168803 PMC7143097

[45] Kelly J Vanhatalo A Bailey SJ Wylie LJ Tucker C List S. Dietary nitrate supplementation: effects on plasma nitrite and pulmonary O2 uptake dynamics during exercise in hypoxia and normoxia. Am J Physiol Regul Integr Comp Physiol. (2014) 307:R920–30. 10.1152/ajpregu.00068.2014 25009219

[46] Pinna M Roberto S Milia R Marongiu E Olla S Loi A. Effect of beetroot juice supplementation on aerobic response during swimming. Nutrients. (2014) 6:605–15. 10.3390/nu6020605 24481133 PMC3942720

[47] Arnold JT Oliver SJ Lewis-Jones TM Wylie LJ Macdonald JH. Beetroot juice does not enhance altitude running performance in well-trained athletes. Appl Physiol Nutr Metab. (2015) 40:590–5. 10.1139/apnm-2014-0470 25942474

[48] MacLeod KE Nugent SF Barr SI Koehle MS Sporer BC MacInnis MJ. Acute beetroot juice supplementation does not improve cycling performance in normoxia or moderate hypoxia. Int J Sport Nutr Exerc Metab. (2015) 25:359–66. 10.1123/ijsnem.2014-0129 25811674

[49] Whitfield J Ludzki A Heigenhauser GJ Senden JM Verdijk LB van Loon LJ. Beetroot juice supplementation reduces whole body oxygen consumption but does not improve indices of mitochondrial efficiency in human skeletal muscle. J Physiol. (2016) 594:421–35. 10.1113/JP270844 26457670 PMC4713742

[50] Betteridge S Bescós R Martorell M Pons A Garnham AP Stathis CC. No effect of acute beetroot juice ingestion on oxygen consumption, glucose kinetics, or skeletal muscle metabolism during submaximal exercise in males. J Appl Physiol. (2016) 120:391–8. 10.1152/japplphysiol.00658.2015 26635348

[51] Thompson C Wylie LJ Blackwell JR Fulford J Black MI Kelly J. Influence of dietary nitrate supplementation on physiological and muscle metabolic adaptations to sprint interval training. J Appl Physiol. (2017) 122:642–52. 10.1152/japplphysiol.00909.2016 27909231 PMC5401949

[52] Santana J Madureira D de França E Rossi F Rodrigues B Fukushima A. Nitrate supplementation combined with a running training program improved time-trial performance in recreationally trained runners. Sports. (2019) 7:120. 10.3390/sports7050120 31117193 PMC6571712

[53] Pawlak-Chaouch M Boissière J Munyaneza D Gamelin FX Cuvelier G Berthoin S. Beetroot juice does not enhance supramaximal intermittent exercise performance in elite endurance athletes. J Am Coll Nutr. (2019) 38:729–38. 10.1080/07315724.2019.1601601 31084516

[54] Wickham KA McCarthy DG Pereira JM Cervone DT Verdijk LB van Loon LJ. No effect of beetroot juice supplementation on exercise economy and performance in recreationally active females despite increased torque production. Physiol Rep. (2019) 7:e13982. 10.14814/phy2.13982 30653856 PMC6336290

[55] Behrens CE Jr Ahmed K Ricart K Linder B Fernández J Bertrand B. Acute beetroot juice supplementation improves exercise tolerance and cycling efficiency in adults with obesity. Physiol Rep. (2020) 8:e14574. 10.14814/phy2.14574 33063953 PMC7556310

[56] Roth T. Benefits of Beetroot Supplementation on Maximal Exercise, Blood Pressure, and the Redox State of Blood. Long Beach, CA: California State University? (2015).

[57] Kozlowska L Mizera O Gromadzińska J Janasik B Mikołajewska K Mróz A. Changes in oxidative stress, inflammation, and muscle damage markers following diet and beetroot juice supplementation in elite fencers. Antioxidants. (2020) 9:571. 10.3390/antiox9070571 32630279 PMC7402086

[58] Ferguson SK Hirai DM Copp SW Holdsworth CT Allen JD Jones AM. Impact of dietary nitrate supplementation via beetroot juice on exercising muscle vascular control in rats. J Physiol. (2012) 591:547–57. 10.1113/jphysiol.2012.243121 23070702 PMC3577528

[59] Hernández A Schiffer TA Ivarsson N Cheng AJ Bruton JD Lundberg JO. Dietary nitrate increases tetanic [Ca^2+^]i and contractile force in mouse fast-twitch muscle. J Physiol. (2012) 590:3575–83. 10.1113/jphysiol.2012.232777 22687611 PMC3547271

[60] Andrade FH Reid MB Allen DG Westerblad H. Effect of hydrogen peroxide and dithiothreitol on contractile function of single skeletal muscle fibres from the mouse. J Physiol. (1998) 509:565–75. 10.1111/j.1469-7793.1998.565bn.x 9575304 PMC2230964

[61] Mora-Rodriguez R Pallarés JG López-Gullón JM López-Samanes Á Fernández-Elías VE Ortega JF. Improvements on neuromuscular performance with caffeine ingestion depend on the time-of-day. J Sci Med Sport. (2015) 18:338–42. 10.1016/j.jsams.2014.04.010 24816164

[62] Bailey SJ Gandra PG Jones AM Hogan MC Nogueira L. Incubation with sodium nitrite attenuates fatigue development in intact single mouse fibres at physiological. J Physiol. (2019) 597:5429–43. 10.1113/JP278494 31541562 PMC6938685

[63] Nyakayiru J Kouw IW Cermak NM Senden JM Van Loon LJC Verdijk LB. Sodium nitrate ingestion increases skeletal muscle nitrate content in humans. J Appl Physiol. (2017) 123:637–44. 10.1152/japplphysiol.01036.2016 28663382

[64] Stamler JS Meissner G. Physiology of nitric oxide in skeletal muscle. Physiol Rev. (2001) 81:209–37. 10.1152/physrev.2001.81.1.209 11152758

[65] Stamler JS. Redox signaling: nitrosylation and related target interactions of nitric oxide. Cell. (1994) 78:931–6. 10.1016/0092-8674(94)90269-0 7923362

[66] Evangelista AM Rao VS Filo AR Marozkina NV Doctor A Jones DR. Direct regulation of striated muscle myosins by nitric oxide and endogenous nitrosothiols. PLoS ONE. (2010) 5:e11209. 10.1371/journal.pone.0011209 20585450 PMC2887846

[67] Nogueira L Figueiredo-Freitas C Casimiro-Lopes G Magdesian MH Assreuy J Sorenson MM. Myosin is reversibly inhibited by S-nitrosylation. Biochem J. (2009) 424:221–31. 10.1042/BJ20091144 19747166

[68] Dutka TL Mollica JP Lamboley CR Weerakkody VC Greening DW Posterino GS. S-nitrosylation and S-glutathionylation of Cys134 on troponin I have opposing competitive actions on Ca2+ sensitivity in rat fast-twitch muscle fibers. Am J Physiol Physiol. (2017) 312:C316–27. 10.1152/ajpcell.00334.2016 27974300

[69] Ishii T Sunami O Saitoh N Nishio H Takeuchi T Hata F. Inhibition of skeletal muscle sarcoplasmic reticulum Ca2+-ATPase by nitric oxide. FEBS Lett. (1998) 440:218–22. 10.1016/S0014-5793(98)01460-4 9862458

[70] Eu JP Sun J Xu L Stamler JS Meissner G. The skeletal muscle calcium release channel: coupled O2 sensor and NO signaling functions. Cell. (2000) 102:499–509. 10.1016/S0092-8674(00)00054-4 10966111

[71] Stoyanovsky D Murphy T Anno PR Kim YM Salama G. Nitric oxide activates skeletal and cardiac ryanodine receptors. Cell Calcium. (1997) 21:19–29. 10.1016/S0143-4160(97)90093-2 9056074

[72] Gould N Doulias PT Tenopoulou M Raju K Ischiropoulos H. Regulation of protein function and signaling by reversible cysteine S-Nitrosylation. J Biol Chem. (2013) 288:26473–79. 10.1074/jbc.R113.460261 23861393 PMC3772194

[73] -Coggan AR Broadstreet SR Mikhalkova D Bole I Leibowitz JL Kadkhodayan A. Dietary nitrate-induced increases in human muscle power: high versus low responders. Physiol Rep. (2018) 6:e13575. 10.14814/phy2.13575 29368802 PMC5789728

[74] Allen DG Lamb GD Westerblad H. Skeletal muscle fatigue: cellular mechanisms. Physiol Rev. (2008) 88:287–332. 10.1152/physrev.00015.2007 18195089

[75] Trump ME Heigenhauser GJ Putman CT Spriet LL. Importance of muscle phosphocreatine during intermittent maximal cycling. J Appl Physiol. (1996) 80:1574–80. 10.1152/jappl.1996.80.5.1574 8727542

[76] Vanhatalo A Fulford J Bailey SJ Blackwell JR Winyard PG Jones AM. Dietary nitrate reduces muscle metabolic perturbation and improves exercise tolerance in hypoxia. J Physiol. (2011) 589:5517–28. 10.1113/jphysiol.2011.216341 21911616 PMC3240888

[77] Bogdanis GC Nevill ME Boobis LH Lakomy HK Nevill A. Recovery of power output and muscle metabolites following 30 s of maximal sprint cycling in man. J Physiol. (1995) 482:467–80. 10.1113/jphysiol.1995.sp020533 7714837 PMC1157744

[78] Bogdanis GC Nevill ME Boobis LH Lakomy HK. Contribution of phosphocreatine and aerobic metabolism to energy supply during repeated sprint exercise. J Appl Physiol. (1996) 80:876–84. 10.1152/jappl.1996.80.3.876 8964751

[79] Gaitanos GC Williams C Boobis LH Brooks S. Human muscle metabolism during intermittent maximal exercise. J Appl Physiol. (1993) 75:712–9. 10.1152/jappl.1993.75.2.712 8226473

[80] Coffey VG Hawley JA. Concurrent exercise training: do opposites distract? J Physiol. (2016) 595:2883–96. 10.1113/JP272270 27506998 PMC5407958

[81] Fyfe JJ Bishop D Stepto NK. Interference between concurrent resistance and endurance exercise: molecular bases and the role of individual training variables. Sports Med. (2014) 44:743–62. 10.1007/s40279-014-0162-1 24728927

[82] Pride CK Mo L Quesnelle KM Dagda RK Murillo D Geary L. Nitrite activates protein kinase A in normoxia to mediate mitochondrial fusion and tolerance to ischaemia/reperfusion. Cardiovasc Res. (2013) 101:57–68. 10.1093/cvr/cvt224 24081164 PMC3868348

[83] Miyazaki M Moriya N Takemasa T. Transient activation of mTORC1 signaling in skeletal muscle is independent of Akt1 regulation. Physiol Rep. (2020) 8:e14599. 10.14814/phy2.14599 33038070 PMC7547586

[84] Goodman CA. Role of mTORC1 in mechanically induced increases in translation and skeletal muscle mass. J Appl Physiol. (2019) 127:581–90. 10.1152/japplphysiol.01011.2018 30676865

[85] Jones AM Ferguson SK Bailey SJ Vanhatalo A Poole DC. Fiber type- specific effects of dietary nitrate. Exerc Sport Sci. (2016) 44:53–60. 10.1249/JES.0000000000000074 26829247

[86] McMahon S Jenkins D. Factors affecting the rate of phosphocreatine resynthesis following intense exercise. Sports Med. (2002) 32:761–84. 10.2165/00007256-200232120-00002 12238940

[87] Siervo M Lara J Ogbonmwan I Mathers JC. Inorganic nitrate and beetroot juice supplementation reduces blood pressure in adults: a systematic review and meta-analysis. J Nutr. (2013) 143:818–26. 10.3945/jn.112.170233 23596162

[88] Barclay CJ Woledge RC Curtin NA. Energy turnover for Ca_2_ cycling in skeletal muscle. J Muscle Res Cell Motil. (2007) 28:259274. 10.1007/s10974-007-9116-7 17882515

[89] Chance B Williams GR. Respiratory enzymes in oxidative phosphorylation. I. Kinetics of oxygen utilization. J Biol Chem. (1955) 217:383393. 10.1016/S0021-9258(19)57190-3 13271402

[90] Mahler M. First-order kinetics of muscle oxygen consumption, and equivalent proportionality between QO2 and phosphorylcreatine level. implications for the control of respiration. J Gen Physiol. (1985) 86:135165. 10.1085/jgp.86.1.135 4031824 PMC2228776

[91] Bose S French S Evans FJ Joubert F Balaban RS. Metabolic network control of oxidative phosphorylation: multiple roles of inorganic phosphate. J Biol Chem. (2003) 278:3915539165. 10.1074/jbc.M306409200 12871940

[92] Bailey SJ Winyard P Vanhatalo A Blackwell JR DiMenna FJ Wilkerson DP. Dietary nitrate supplementation reduces the O2 cost of low-intensity exercise and enhances tolerance to high-intensity exercise in humans. J Applied Physiol. (2009) 107:1144–55. 10.1152/japplphysiol.00722.2009 19661447

[93] Hardie DG. AMPK-sensing energy while talking to other signaling pathways. Cell Metab. (2014) 20:939–52. 10.1016/j.cmet.2014.09.013 25448702 PMC5693325

[94] Wadley GD Lee-Young RS Canny BJ Wasuntarawat C Chen ZP Hargreaves M. Effect of exercise intensity and hypoxia on skeletal muscle AMPK signaling and substrate metabolism in humans. Am J Physiol Endocrinol Metab. (2006) 290:E694–702. 10.1152/ajpendo.00464.2005 16263768

[95] Bonilla Ocampo DA Paipilla AF Marín E Vargas-Molina S Petro JL Pérez-Idárraga A. Dietary nitrate from beetroot juice for hypertension: a systematic review. Biomolecules. (2018) 8:134. 10.3390/biom8040134 30400267 PMC6316347

[96] Raubenheimer K Hickey D Leveritt M Fassett R Ortiz de Zevallos Munoz J Allen JD. Acute effects of nitrate-rich beetroot juice on blood pressure, hemostasis and vascular inflammation markers in healthy older adults: a randomized, placebo-controlled crossover study. Nutrients. (2017) 9:1270. 10.3390/nu9111270 29165355 PMC5707742

[97] Hoon MW Jones AM Johnson NA Blackwell JR Broad EM Lundy B. The effect of variable doses of inorganic nitrate-rich beetroot juice on simulated 2000-m rowing performance in trained athletes. Int J Sports Physiol Perform. (2014) 9:615–20. 10.1123/ijspp.2013-0207 24085341

[98] Martí JG Morales AM Bosch MP Cid AV Ferrari MR. El efecto del zumo de remolacha sobre la presión arterial y el ejercicio físico: revisión sistemática. Rev Esp Nutr Com. (2015) 21:20–9. 10.14642/RENC.2015.21.2.5099

[99] Axe DD Bailey JE. Transport of lactate and acetate through the energized cytoplasmic membrane of *Escherichia coli*. Biotechnol Bioeng. (1995) 47:8–19. 10.1002/bit.260470103 18623362

[100] Cermak NM Gibala MJ van Loon LJC. Nitrate supplementation's improvement of 10-km time-trial performance in trained cyclists. Int J Sport Nutr Exerc Metab. (2012) 22:64–71. 10.1123/ijsnem.22.1.64 22248502

[101] Shannon OM Barlow MJ Duckworth L Williams E Wort G Woods D. Dietary nitrate supplementation enhances short but no longer duration running time-trial performance. Eur J Appl Physiol. (2017) 117:775–85. 10.1007/s00421-017-3580-6 28251402

[102] Husmann F Bruhn S Mittlmeier T Zschorlich V Behrens M. Dietary nitrate supplementation improves exercise tolerance by reducing muscle fatigue and perceptual responses. Front Physiol. (2019) 10:404. 10.3389/fphys.2019.00404 31068827 PMC6491676

[103] Poulet JFA Hedwig B. New insights into corollary discharges mediated by identified neural pathways. Trends Neurosci. (2007) 30:14–21. 10.1016/j.tins.2006.11.005 17137642

[104] DeMorree HM Klein C Marcora SM. Perception of effort reflects central motor command during movement execution. Psychophysiology. (2012) 49:1242–53. 10.1111/j.1469-8986.2012.01399.x 22725828

[105] Rooks CR Thom NJ McCully KK Dishman RK. Effects of incremental exercise on cerebral oxygenation measured by near-infrared spectroscopy: a systematic review. Prog Neurobiol. (2010) 92:134–50. 10.1016/j.pneurobio.2010.06.002 20542078

[106] Wylie LJ Park JW Vanhatalo A Kadach S Black MI Stoyanov Z. Human skeletal muscle nitrate store: influence of dietary nitrate supplementation and exercise. J Physiol. (2019) 597:5565–76. 10.1113/JP278076 31350908 PMC9358602

[107] Srihirun S Park JW Teng R Sawaengdee W Piknova B Schechter AN. Nitrate uptake and metabolism in human skeletal muscle cell cultures. Nitric Oxide. (2020) 94:1–8. 10.1016/j.niox.2019.10.005 31604144 PMC7341890

[108] Rettori V Belova N Dees WL Nyberg CL Gimeno M McCann SM. Role of nitric oxide in the control of luteinizing hormone-releasing hormone release *in vivo* and *in vitro*. Proc Natl Acad Sci USA. (1993) 90:10130–4. 10.1073/pnas.90.21.10130 7694282 PMC47727

[109] Lowenstein CJ Dinerman JL Snyder SH. Nitric oxide: a physiologic messenger. Ann Intern Med. (1994) 120:227–37. 10.7326/0003-4819-120-3-199402010-00009 8273987

[110] Tsuchiya T Kishimoto J Koyama J Ozawa T. Modulatory effect of L-NAME, a specific nitric oxide synthase (NOS) inhibitor, on stress-induced changes in plasma adrenocorticotropic hormone (ACTH) and corticosterone levels in rats: physiological significance of stress-induced NOS activation in hypothalamic–pituitary–adrenal axis. Brain Res. (1997) 776:68–74. 10.1016/S0006-8993(97)00942-6 9439797

[111] Di Luigi L Baldari C Sgro P Pietro EG Gallotta MC Bianchini S. The type 5 phosphodiesterase inhibitor tadalafil influences salivary cortisol, testosterone, and dehydroepiandrosterone sulphate responses to maximal exercise in healthy men. J Clin Endocrinol Metab. (2008) 93:3510–4. 10.1210/jc.2008-0847 18559908

[112] VanBruggen MD Hackney AC McMurray RG Ondrak KS. The relationship between serum and salivary cortisol levels in response to different intensities of exercise. Int J Sports Physiol Perform. (2011) 6:396–407. 10.1123/ijspp.6.3.396 21911864

[113] Arlt W Stewart PM. Adrenal corticosteroid biosynthesis, metabolism, and action. Endocrinol Metab Clin N Am. (2005) 34:293–313. 10.1016/j.ecl.2005.01.002 15850843

[114] Dinneen S Alzaid A Miles J Rizza R. Metabolic effects of the nocturnal rise in cortisol on carbohydrate metabolism in normal humans. J Clin Invest. (1993) 92:2283–90. 10.1172/JCI116832 8227343 PMC288409

[115] Berneis K Ninnis R Girard J Frey BM Keller U. Effects of insulin-like growth factor I combined with growth hormone on glucocorticoidinduced whole-body protein catabolism in man. J Clin Endocrinol Metab. (1997) 82:2528–34. 10.1210/jc.82.8.2528 9253329

[116] Djurhuus CB Gravholt CH Nielsen S Mengel A Christiansen JS Schmitz OE. Effects of cortisol on lipolysis and regional interstitial glycerol levels in humans. Am J Physiol Endocrinol Metab. (2002) 283:E172–7. 10.1152/ajpendo.00544.2001 12067858

[117] Valenti S Cuttica CM Fazzuoli L Giordano G Giusti M. Biphasic effect of nitric oxide on testosterone and cyclic GMP production by purified rat Leydig cells cultured *in vitro*. Int J Androl. (1999) 22:336–41. 10.1046/j.1365-2605.1999.00189.x 10509235

[118] Yildiz O Seyrek M Gul H Un I Yildirim V Ozal E. Testosterone relaxes human internal mammary artery *in vitro*. J Cardiovasc Pharmacol. (2005) 45:580–5. 10.1097/01.fjc.0000161400.06704.1e 15897786

[119] Han DH Chae MR Jung JH So I Park JK Lee SW. Effect of testosterone on potassium channel opening in human corporal smooth muscle cells. J Sex Med. (2008) 5:822–32. 10.1111/j.1743-6109.2007.00732.x 18208499

[120] Lorigo M Mariana M Lemos MC Cairrao E. Vascular mechanisms of testosterone: the non-genomic point of view. J Steroid Biochem Mol Biol. (2020) 196:105496. 10.1016/j.jsbmb.2019.105496 31655180

[121] Alderton WK Cooper CE Knowles RG. Nitric oxide synthases: structure, function and inhibition. Biochem J. (2001) 357:593. 10.1042/0264-6021:3570593 11463332 PMC1221991

[122] Lancaster JR. A tutorial on the diffusibility and reactivity of free nitric oxide. Nitric Oxide. (1997) 1:18–30. 10.1006/niox.1996.0112 9701041

[123] Schuman E Madison D. A requirement for the intercellular messenger nitric oxide in long-term potentiation. Science. (1991) 254:1503–06. 10.1126/science.1720572 1720572

[124] Lonart G Wang J Johnson KM. Nitric oxide induces neurotransmitter release from hippocampal slices. Eur J Pharmacol. (1992) 220:271–2. 10.1016/0014-2999(92)90759-W 1425999

[125] Lorrain DS Hull EM. Nitric oxide increases dopamine and serotonin release in the medial preoptic area. NeuroReport. (1993) 5:87–9. 10.1097/00001756-199310000-00024 8280866

[126] Guevara-Guzman R Emson PC Kendrick KM. Modulation of *in vivo* striatal transmitter release by nitric oxide and cyclic GMP. J Neurochem. (1994) 62:807–10. 10.1046/j.1471-4159.1994.62020807.x 7905029

[127] Singewald N Kaehler ST Hemeida R Philippu A. Influence of excitatory amino acids on basal and sensory stimuli-induced release of 5-HT in the locus coeruleus. Br J Pharmacol. (1998) 123:746–52. 10.1038/sj.bjp.0701656 9517395 PMC1565214

[128] Heijnen S Hommel B Kibele A Colzato LS. Neuromodulation of aerobic exercise—a review. Front Psychol. (2016) 6:1890. 10.3389/fpsyg.2015.01890 26779053 PMC4703784

[129] Yabut JM Crane JD Green AE Keating DJ Khan WI Steinberg GR. Emerging roles for serotonin in regulating metabolism: new implications for an ancient molecule. Endocr Rev. (2019) 40:1092–107. 10.1210/er.2018-00283 30901029 PMC6624793

[130] Molinoff PB Axelrod J. Biochemistry of Catecholamines. Annu Rev Biochem. (1971) 40:465–500. 10.1146/annurev.bi.40.070171.002341 4399447

[131] Schwarz PB Peever JH. Dopamine triggers skeletal muscle tone by activating D1-like receptors on somatic motoneurons. J Neurophysiol. (2011) 106:1299–309. 10.1152/jn.00230.2011 21653722

[132] Brenner-Lavie H Klein E Zuk R Gazawi H Ljubuncic P Ben-Shachar D. Dopamine modulates mitochondrial function in viable SH-SY5Y cells possibly via its interaction with complex I: relevance to dopamine pathology in schizophrenia. BBA-BIOENERGETICS. (2008) 1777:173–85. 10.1016/j.bbabio.2007.10.006 17996721

[133] Fernstrom JD Fernstrom MH. Tyrosine, phenylalanine, and catecholamine synthesis and function in the brain. J Nutr. (2007) 137:1539S–47S. 10.1093/jn/137.6.1539S 17513421

[134] Bruno RM Ghiadoni L Seravalle G Dell'oro R Taddei S Grassi G. Sympathetic regulation of vascular function in health and disease. Front Physiol. (2012) 3:284. 10.3389/fphys.2012.00284 22934037 PMC3429057

[135] Hirooka Y Kishi T Sakai K Takeshita A Sunagawa K. Imbalance of central nitric oxide and reactive oxygen species in the regulation of sympathetic activity and neural mechanisms of hypertension. Am J Physiol Regul Integr Comp Physiol. (2011) 300:R818–26. 10.1152/ajpregu.00426.2010 21289238

[136] Patel KP Li YF Hirooka Y. Role of nitric oxide in central sympathetic outflow. Exp Biol Med. (2001) 226:814–24. 10.1177/153537020122600902 11568303

[137] Presley TD Morgan AR Bechtold E Clodfelter W Dove RW Jennings JM. Acute effect of a high nitrate diet on brain perfusion in older adults. Nitric Oxide. (2011) 24:34–42. 10.1016/j.niox.2010.10.002 20951824 PMC3018552

[138] Thomas GD Victor RG. Nitric oxide mediates contraction-induced attenuation of sympathetic vasoconstriction in rat skeletal muscle. J Physiol. (1998) 506:817–26. 10.1111/j.1469-7793.1998.817bv.x 9503340 PMC2230749

[139] Bada A Svendsen J Secher N Saltin B Mortensen S. Peripheral vasodilatation determines cardiac output in exercising humans: insight from atrial pacing. J Physiol. (2012) 590:2051–60. 10.1113/jphysiol.2011.225334 22351638 PMC3573320

[140] Duarte J Francisco V Perez-Vizcaino F. Modulation of nitric oxide by flavonoids. Food Funct. (2014) 5:1653–68. 10.1039/C4FO00144C 24901042

[141] Schroeter H Bahia P Spencer JP Sheppard O Rattray M Cadenas E. (-) Epicatechin stimulates ERK-dependent cyclic AMP response element activity and up-regulates GluR2 in cortical neurons. J Neurochem. (2007) 101:1596–606. 10.1111/j.1471-4159.2006.04434.x 17298385

[142] Vauzour D Vafeiadou K Rodriguez-Mateos A Rendeiro C Spencer JP. The neuroprotective potential of flavonoids: a multiplicity of effects. Genes Nutr. (2008) 3:115–26. 10.1007/s12263-008-0091-4 18937002 PMC2593006

[143] Rimer EG Peterson LR Coggan AR Martin JC, performance. Acute dietary nitrate supplementation increases maximal cycling power in athletes. Int J Sports Physiol Perform. (2016) 11:715. 10.1123/ijspp.2015-0533 26641379 PMC4889556

[144] Clementi E Nisoli E. Nitric oxide and mitochondrial biogenesis: a key to long-term regulation of cellular metabolism. Comp Biochem Physiol A Mol Integr Physiol. (2005) 142:102–10. 10.1016/j.cbpb.2005.04.022 16091305

[145] Martins KJ St-Louis M Murdoch GK MacLean IM McDonald P Dixon WT. Nitric oxide synthase inhibition prevents activity-induced calcineurin-NFATc1 signalling and fast- to-slow skeletal muscle fibre type conversions. J Physiol. (2012) 590:1427–42. 10.1113/jphysiol.2011.223370 22219342 PMC3382332

[146] De Smet S Van Thienen R Deldicque L James R Sale C Bishop DJ. Nitrate intake promotes shift in muscle fiber type composition during sprint interval training in hypoxia. Front Physiol. (2016) 7:233. 10.3389/fphys.2016.00233 27378942 PMC4906611

[147] Sarti P Arese M Forte E Giuffre A Mastronicola D. Mitochondria and nitric oxide: chemistry and pathophysiology. Adv Exp Med Biol. (2012) 942:75–92. 10.1007/978-94-007-2869-1_4 22399419

[148] Coggan AR Peterson LR. Dietary nitrate enhances the contractile properties of human skeletal muscle. Exerc Sport Sci Rev. (2018) 46:254–61. 10.1249/JES.0000000000000167 30001275 PMC6138552

[149] Brown GC Cooper CE. Nanomolar concentrations of nitric oxide reversibly inhibit synaptosomal respiration by competing with oxygen at cytochrome oxidase. FEBS Lett. (1994) 356:295–8. 10.1016/0014-5793(94)01290-3 7805858

[150] Cleeter MWJ Cooper JM Darley-Usmar VM Moncada SA Schapira AHV. Reversible inhibition of cytochrome c oxidase, the terminal enzyme of the mitochondrial respiratory chain, by nitric oxide: implications for neurodegenerative diseases. FEBS Lett. (1994) 345:50–4. 10.1016/0014-5793(94)00424-2 8194600

[151] Shen W Xu X Ochoa M Zhao G Wolin MS Hintze TH. Role of nitric oxide in the regulation of oxygen consumption in conscious dogs. Circ Res. (1994) 75:1086–95. 10.1161/01.RES.75.6.1086 7525103

[152] Heinonen I Bengt S Jukka K Sipilä HT Vesa O Pirjo N. Skeletal muscle blood flow and oxygen uptake at rest and during exercise in humans: a PET study with nitric oxide and cyclooxygenase inhibition. Am J Physiol Heart Circ Physiol. (2011) 300:H1510–7. 10.1152/ajpheart.00996.2010 21257921

[153] Antunes F Boveris A Cadenas E. On the mechanism and biology of cytochrome oxidase inhibition by nitric oxide. Proc Natl Acad Sci USA. (2004) 101:16774–9. 10.1073/pnas.0405368101 15546991 PMC534717

[154] Rossignol R Faustin B Rocher C Malgat M Mazat JP Letellier T. Mitochondrial threshold effects. Biochem J. (2003) 370:751–62. 10.1042/bj20021594 12467494 PMC1223225

[155] Clerc P Rigoulet M Leverve X Fontaine E. Nitric oxide increases oxidative phosphorylation efficiency. J Bioenerg Biomembr. (2007) 39:158–66. 10.1007/s10863-007-9074-1 17447126

[156] Kadenbach B Hüttemann M Arnold S Lee I Bender E. Mitochondrial energy metabolism is regulated via nuclear-coded subunits of cytochrome c oxidase. Free Radic Biol Med. (2000) 29:211–21. 10.1016/S0891-5849(00)00305-1 11035249

[157] Shen W Tian R Saupe KW Spindler M Ingwall JS. Endogenous nitric oxide enhances coupling between O_2_ consumption and ATP synthesis in guinea pig hearts. Am J Physiol Heart Circ Physiol. (2001) 281:H838–46. 10.1152/ajpheart.2001.281.2.H838 11454589

[158] Erusalimsky JD Moncada S. Nitric oxide and mitochondrial signaling: from physiology to pathophysiology. Arterioscler Thromb Vasc Biol. (2007) 27:2524–31. 10.1161/ATVBAHA.107.151167 17885213

[159] Larsen FJ Schiffer TA Weitzberg E Lundberg JO. Regulation of mitochondrial function and energetics by reactive nitrogen oxides. Free Radic Biol Med. (2012) 53:1919–28. 10.1016/j.freeradbiomed.2012.08.580 22989554

[160] Garland CJ Hiley CR Dora KA. EDHF: spreading the influence of the endothelium. Br J Pharmacol. (2011) 164:839–52. 10.1111/j.1476-5381.2010.01148.x 21133895 PMC3195909

[161] Reid K. Performance food: promoting foods with a functional benefit in sports performance. Nutr Bull. (2013) 38:429–37. 10.1111/nbu.12065

[162] Bugger H Abel ED. Mitochondria in the diabetic heart. Cardiovasc Res. (2010) 88:229–40. 10.1093/cvr/cvq239 20639213 PMC2952534

[163] Wylie LJ Zevallos JO Isidore T Nyman L Vanhatalo A Bailey SJ. Dose-dependent effects of dietary nitrate on the oxygen cost of moderate-intensity exercise: acute vs. chronic supplementation. Nitric Oxide. (2016) 57:30–9. 10.1016/j.niox.2016.04.004 27093910

[164] Campos HO Drummond LR Rodrigues QT Machado FS Pires W Wanner SP. Nitrate supplementation improves physical performance specifically in non-athletes during prolonged open-ended tests: a systematic review and meta-analysis. Br J Nutr. (2018) 119:636–57. 10.1017/S0007114518000132 29553034

[165] Georgiev VG Weber J Kneschke EM Denev PN Bley T Pavlov AI. Antioxidant activity and phenolic content of betalain extracts from intact plants and hairy root cultures of the red beetroot beta vulgaris Cv. Detroit Dark Red. Plant Foods Hum Nutr. (2010) 65:105–11. 10.1007/s11130-010-0156-6 20195764

[166] Kujala T Vienola M Klika K Loponen J Pihlaja K. Betalain and phenolic compositions of four beetroot (Beta Vulgaris) cultivars. Eur Food Res Technol. (2002) 214:505–10. 10.1007/s00217-001-0478-6

[167] Wootton-Beard PC Ryan L. A beetroot juice shot is a significant and convenient source of bioaccessible antioxidants. J Funct Foods. (2011) 3:329–34. 10.1016/j.jff.2011.05.007

[168] Lee CH Wettasinghe M Bolling BW Ji LL Parkin KL. Betalains, Phase II enzyme-inducing components from red beetroot (*Beta vulgaris* L.) Extracts. Nutr Cancer. (2005) 53:91–103. 10.1207/s15327914nc5301_11 16351511

[169] Vulic JJ Cebovic TN Canadanovic-Brunet JM Cetkovic GS Canadanovic VM Djilas SM. *In vivo* and *in vitro* antioxidant effects of beetroot pomace extracts. J Funct Foods. (2014) 6:168–75. 10.1016/j.jff.2013.10.003

[170] Zielińska-Przyjemska M Olejnik A Dobrowolska-Zachwieja A Grajek W. *In vitro* effects of beetroot juice and chips on oxidative metabolism and apoptosis in neutrophils from obese individuals. Phytother Res. (2009) 23:49–55. 10.1002/ptr.2535 18814207

[171] Pavlov A Georgiev V Ilieva M. Betalain biosynthesis by red beet (Beta Vulgaris L.) hairy root culture. Process Biochem. (2005) 40:1531–3. 10.1016/j.procbio.2004.01.001

[172] Tesoriere L Allegra M Butera D Livrea MA. Absorption, excretion, and distribution of dietary antioxidant betalains in LDLs: potential health effects of betalains in humans. Am J Clin Nutr. (2004) 80:941–5. 10.1093/ajcn/80.4.941 15447903

[173] Vidal PJ López-Nicolás JM Gandía-Herrero F García-Carmona F. Inactivation of lipoxygenase and cyclooxygenase by natural betalains and semi-synthetic analogues. Food Chem. (2014) 154:246–54. 10.1016/j.foodchem.2014.01.014 24518339

[174] Manach C Williamson G Morand C Scalbert A Rémésy C. Bioavailability and bioefficacy of polyphenols in humans. i. review of 97 bioavailability studies. Am J Clin Nutr. (2005) 81:230S–42S. 10.1093/ajcn/81.1.230S 15640486

[175] Jones AM. Influence of dietary nitrate on the physiological determinants of exercise performance: a critical review. Appl Physiol Nutr Metab. (2014) 39:1019–28. 10.1139/apnm-2014-0036 25068792

[176] Lundberg JO Carlström M Larsen FJ Weitzberg E. Roles of dietary inorganic nitrate in cardiovascular health and disease. Cardiovasc Res. (2011) 89:525–32. 10.1093/cvr/cvq325 20937740

[177] Wink DA Hines HB Cheng RYS Switzer CH Flores-Santana W Vitek MP. Nitric oxide and redox mechanisms in the immune response. J Leukoc Biol. (2011) 89:873–91. 10.1189/jlb.1010550 21233414 PMC3100761

[178] Kujawska M Ignatowicz E Murias M Ewertowska M Mikołajczyk K Jodynis-Liebert J. Protective effect of red beetroot against carbon tetrachloride- and N-nitrosodiethylamineinduced oxidative stress in rats. J Agric Food Chem. (2009) 57:2570–5. 10.1021/jf803315d 19292473

[179] Winkler C Wirleitner B Schroecksnadel K Schennach H Fuchs D. *In vitro* effects of beetroot juice on stimulated and unstimulated peripheral blood mononuclear cells. Am J Biochem Biotechnol. (2005) 1:180. 10.3844/ajbbsp.2005.180.185

[180] Wootton-Beard PC Moran A Ryan L. Stability of the total antioxidant capacity and total polyphenol content of 23 commercially available vegetable juices before and after *in vitro* digestion measured by FRAP, DPPH, ABTS and Folin-Ciocalteu methods. Food Res Int. (2011) 44:217–24. 10.1016/j.foodres.2010.10.033

[181] Tom C Glyn H Daniel JW Emma JS. The potential benefits of red beetroot supplementation in health and disease. Nutrients. (2015) 7:2801–22. 10.3390/nu7042801 25875121 PMC4425174

[182] Silva DVTD Silva FDO Perrone D Pierucci APTR Conte-Junior CA Alvares TDS. Physicochemical, nutritional, and sensory analyses of a nitrate enriched beetroot gel and its effects on plasmatic nitric oxide and blood pressure. Food Nutr Res. (2016) 60:29909. 10.3402/fnr.v60.29909 26790368 PMC4720688

[183] Esatbeyoglu T Wagner AE Schini-Kerth VB Rimbach G. Betanin-a food colorant with biological activity. Mol Nutr Food Res. (2015) 59:36–47. 10.1002/mnfr.201400484 25178819

[184] Fiordelisi A Iaccarino G Morisco C Coscioni E Sorriento D. NF kappa B is a key player in the crosstalk between inflammation and cardiovascular diseases. Int J Mol Sci. (2019) 20:1599. 10.3390/ijms20071599 30935055 PMC6480579

[185] Satta S Mahmoud AM Wilkinson FL Alexander YM White SJ. The role of Nrf2 in cardiovascular function and disease. Oxid Med Cell Longev. (2017) 2017:9237263. 10.1155/2017/9237263 29104732 PMC5618775

[186] Silva DVTD Baião DDS Ferreira VF Paschoalin VMF. Betanin as a multipath oxidative stress and inflammation modulator: a beetroot pigment with protective effects on cardiovascular disease pathogenesis. Crit Rev Food Sci Nutr. (2020) 60:1–16. 10.1080/10408398.2020.1822277 32997545

[187] Kumar S Pandey AK. Chemistry and biological activities of flavonoids: an overview. Sci World J. (2013) 2013:162750. 10.1155/2013/162750 24470791 PMC3891543

[188] Bors W Michel C Stettmaier K. Antioxidant effects of flavonoids. Bio Factors. (1997) 6:399–402. 10.1002/biof.5520060405 9388305

[189] Nijveldt RJ van Nood E van Hoorn DEC Boelens PG van Norren K van Leeuwen PAM. Flavonoids: a review of probable mechanisms of action and potential applications. Am J Clin Nutr. (2001) 74:418–25. 10.1093/ajcn/74.4.418 11566638

[190] Ferriola PC Cody V Middleton E. Protein kinase C inhibition by plant flavonoids: kinetic mechanisms and structure–activity relationships. Biochem Pharmacol. (1989) 38:1617–24. 10.1016/0006-2952(89)90309-2 2730676

[191] Nayik GA Gull A. Antioxidants in Vegetables and Nuts-Properties and Health Benefits. Springer Singapore Pte. Limited (2020). 10.1007/978-981-15-7470-2

[192] Ridderstråle W Saluveer O Carlström M. Jern S, Hrafnkelsdóttir TJ. The impaired fibrinolytic capacity in hypertension is unaffected by acute blood pressure lowering. J Thromb Thrombolysis. (2011) 32:399. 10.1007/s11239-011-0595-4 21614456

[193] Gao X Yang T Liu M Peleli M Zollbrecht C Weitzberg E. NADPH oxidase in the renal microvasculature is a primary target for blood pressure-lowering effects by inorganic nitrate and nitrite. Hypertension. (2015) 65:161–70. 10.1161/HYPERTENSIONAHA.114.04222 25312440

[194] Yang T Zhang XM Tarnawski L Peleli M Zhuge Z Terrando N. Dietary nitrate attenuates renal ischemia-reperfusion injuries by modulation of immune responses and reduction of oxidative stress. Redox biology. (2017) 13:320–30. 10.1016/j.redox.2017.06.002 28623824 PMC5473548

[195] Lundberg JO Gladwin MT Weitzberg E. Strategies to increase nitric oxide signalling in cardiovascular disease. Nat Rev Drug Discov. (2015) 14:623–41. 10.1038/nrd4623 26265312

[196] Carlstrom M Lundberg JO Weitzberg E. Mechanisms underlying blood pressure reduction by dietary inorganic nitrate. Acta Physiol. (2018) 224:e13080. 10.1111/apha.13080 29694703

